# Linking genotype to trophoblast phenotype in preeclampsia and HELLP syndrome associated with *STOX1* genetic variants

**DOI:** 10.1016/j.isci.2024.109260

**Published:** 2024-02-16

**Authors:** Lorenzo Costa, Luis Bermudez-Guzman, Ikram Benouda, Paul Laissue, Adrien Morel, Karen Marcela Jiménez, Thierry Fournier, Laurence Stouvenel, Céline Méhats, Francisco Miralles, Daniel Vaiman

**Affiliations:** 1 Institut Cochin, Team ‘From Gametes To Birth’, INSERM U1016, CNRS UMR8104, Université de Paris, 24 rue du Faubourg St Jacques, 75014 Paris, France; 2 Department of Human Genetics, University of Heidelberg, Heidelberg, Germany; 3 CRUK Cambridge Institute, University of Cambridge, Cambridge, UK; 4 Biopas Laboratoires, Orphan Diseases Unit, BIOPAS GROUP, Bogotá 111111, Colombia; 5 Universidad Del Rosario, School of Medicine and Health Sciences, Center for Research in Genetics and Genomics (CIGGUR), Institute of Translational Medicine (IMT), Bogotá, Colombia; 6 Université Paris Cité, INSERM, UMR-S1139, Pathophysiology & Pharmacotoxicology of the Human Placenta, Pre- & Post-natal Microbiota (3PHM), 75006 Paris, France

**Keywords:** Pregnancy, Human Genetics, Phenotyping, Cell biology, Transcriptomics

## Abstract

Preeclampsia is a major hypertensive pregnancy disorder with a 50% heritability. The first identified gene involved in the disease is STOX1, a transcription factor, whose variant Y153H predisposes to the disease. Two rare mutations were also identified in Colombian women affected by the hemolysis, elevated liver enzyme, low platelet syndrome, a complication of preeclampsia (T188N and R364X). Here, we explore the effects of these variants in trophoblast cell models (BeWo) where STOX1 was previously invalidated. We firstly showed that STOX1 knockout alters response to oxidative stress, cell proliferation, and fusion capacity. Then, we showed that mutant versions of STOX1 trigger alterations in gene profiles, growth, fusion, and oxidative stress management. The results also reveal alterations of the STOX interaction with DNA when the mutations affected the DNA-binding domain of STOX1 (Y153H and T188N). We also reveal here that a major contributor of these effects appears to be the E2F3 transcription factor.

## Highlights

• The KO of STOX1 in BeWo cells induces a higher fusogenic capacity
• STOX1 acts through the regulation of the E2F3 transcription factor
• The R364X mutant has altered oxidative stress, proliferation, and fusion
• hCG secretion is altered in BeWo cells overexpressing STOX1 mutants

## Graphical abstract

**Figure:**
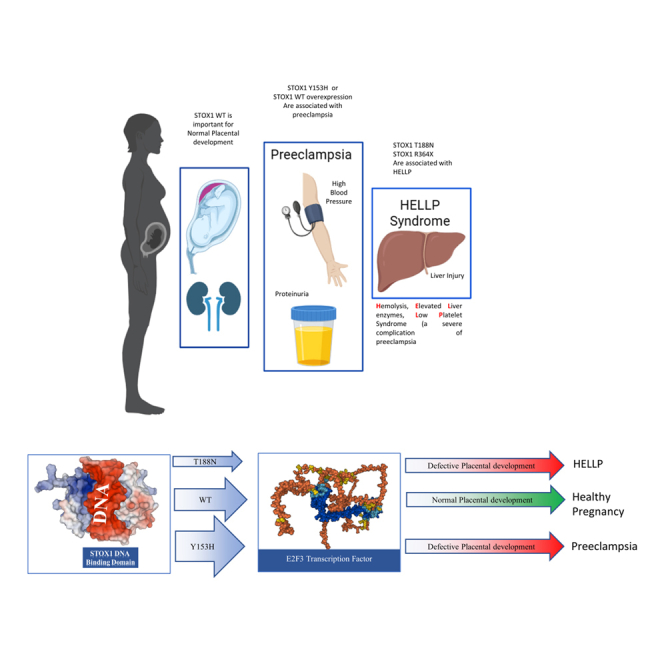

## Introduction

Preeclampsia is a pregnancy-associated disease with maternal symptoms, but of placental origin. In 2005, STOX1 (Storkhead bOX 1) was discovered as the first gene involved in genetic forms of preeclampsia in Dutch families.^1^ Later, two major isoforms of this transcription factor were described, STOX1A (989 amino acids) and STOX1B (229 amino acids), sharing the same DNA-binding domain (DBD), but with different transactivating activities.^2^ STOX1 impacts the expression of numerous genes. This was shown in trophoblast cell lines, such as JEG-3^3^ and BeWo cells,^2^ where overexpression of STOX1 led to the significant deregulation of more than 2,000 genes. In the Dutch families, the missense variant Y153H was found as the predominant variant in STOX1, affecting specifically the DBD of the protein.^1^ Notably, this variant was later found in other preeclamptic populations (namely other Netherlands and Turkey cohorts^4^^,^^5^). Some of the effects of the Y153H variants have previously been characterized, such as trophoblast invasion decrease in the first trimester of pregnancy through upregulation of CTNNA3 (Alpha-T-catenin).^6^

Other potentially deleterious variants of STOX1 have been identified in a whole-exome sequencing study carried out in Colombian patients affected by the HELLP (hemolysis, elevated liver enzyme, low platelet) syndrome, a severe multi-organ complication of preeclampsia. Five out of 79 unrelated HELLP patients were heterozygous carriers of rare deleterious mutations of STOX1 (R364X in one patient and T188N in four patients), absent from 179 controls.^7^ Interestingly, the first mutation is predicted to truncate the protein (364 amino acids) which is closer in length to the STOX1B isoform (229 amino acids) than the full-length isoform, STOX1A. The second mutation, T188N, affects the DBD. Further research showed that the deleterious effect of Y153H (which is in fact a frequent genetic variant present at 38% in the general population) is dependent on a second independent variant affecting the gene encoding the protein NODAL, (R165H), suggesting an epistatic effect.^8^

The involvement of STOX1 in preeclampsia was further substantiated by the creation of transgenic mice overexpressing the human protein in the feto-placental unit, which recapitulate the human symptoms of the disease.^9^ This mouse model was used to study the vascular flow and oxygen load in the preeclamptic placenta,^10^ to follow the maternal endothelial and cardiovascular effects of preeclampsia during the disease,^11^ and the long-term effects upon vascular health.^12^ It was also used to evaluate approaches to treat preeclampsia such as aspirin,^13^ Alpha-1 microglobulin,^14^ and tetrahydrobiopterin.^15^

In the present paper, we propose a functional characterization of the Y153H, T188N, and R364X variants using the BeWo cell model, a trophoblast cell line able to fuse under stimulation of the cyclic AMP (cAMP) cascade with forskolin (FSK) treatment. To achieve this, we first obtained two independent BeWo cell lines where STOX1 was knocked out by the CRISPR-Cas9 technology. Then, starting from these cell lines, we generated new cell lines expressing STOX1A WT, or STOX1A with each of the three variants previously mentioned. The expression profile and physiologic properties of the cell lines were then thoroughly analyzed using transcriptomics, live-cell imaging, secretion of human chorionic gonadotrophin, measures of oxidative stress, and capability to fuse. This leads to insights into the physiopathology of women expressing abnormal STOX1 proteins in the placenta. We also showed by ultrastructural analysis that the two variants affecting the DBD, T188N and Y153H, have opposite effects on the affinity of STOX1 on DNA. Of note, the frequent variant Y153H alters the STOX1 effect through activation of the E2F3 transcription factor and its targets. This variant shifts the role of STOX1 from an equilibrium between trophoblast proliferation and differentiation, toward a pro-proliferative state, thus possibly destabilizing normal placental function. The present study provides new insights into the genotype-phenotype connections in preeclamptic cases related to STOX1 variation.

## Results

### Characterization of STOX1-knockout (KO) BeWo cells

**Figure 1:**
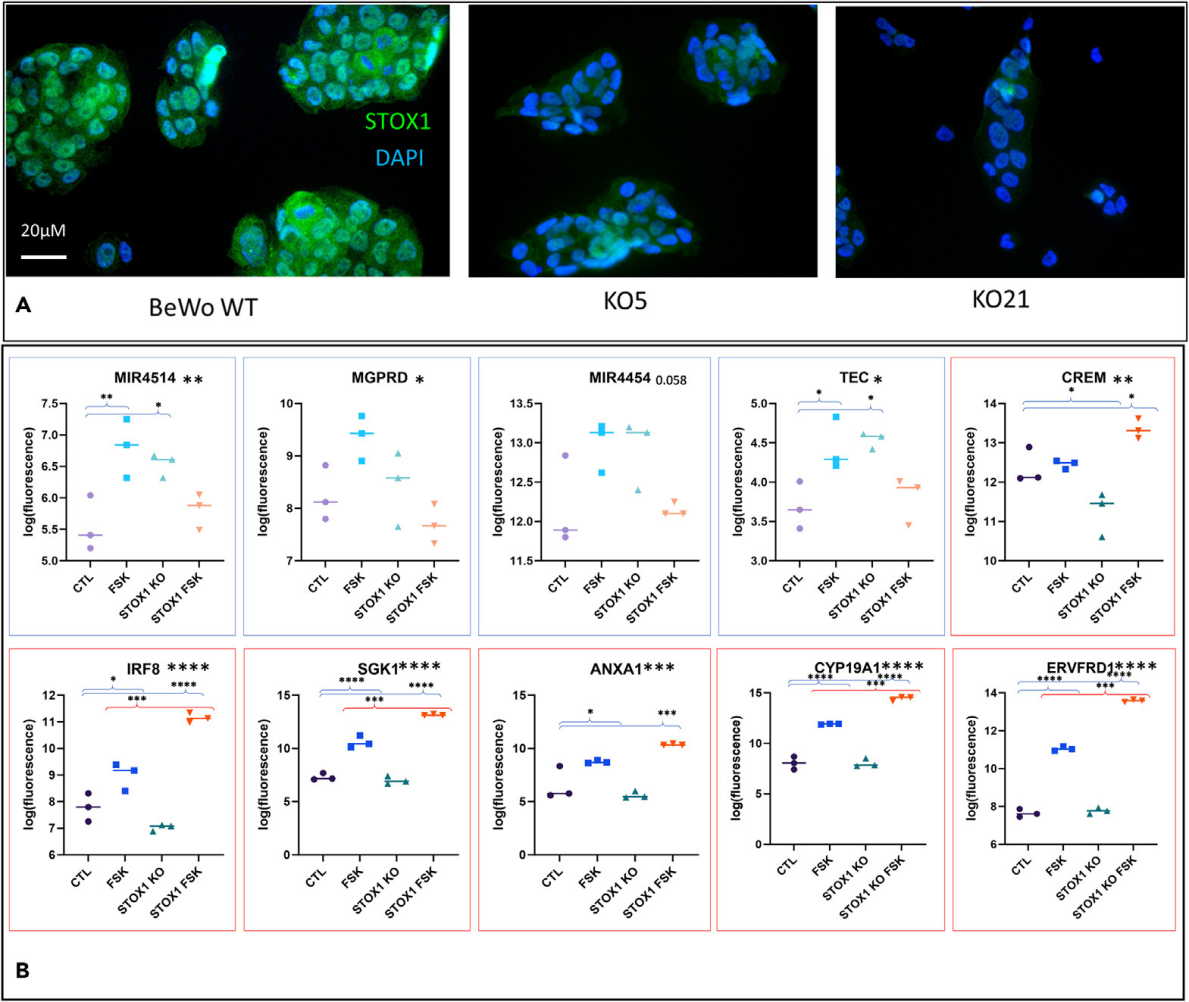
Experimental validation of the STOX1 KO (A) The STOX1 KO cell lines show no staining confirming the absence of STOX1 (PA5-65193, Sigma-Aldrich). The nuclei are labeled with DAPI. (B) A sample of deregulated genes in the STOX1 knockout, in the presence or absence of forskolin (FSK) for three days (20 μM, a condition known to induce cell fusion). The ordinates represent fluorescence measures reported in log 2 base. In blue boxes are presented a sample of genes that display an opposite behavior in the WT and in the KO following treatment. In red boxes are presented a sample of genes for which the FSK effect is amplified in the STOX1 KO cells. The stars near the gene name are through ANOVA one-way analysis, followed by Tukey’s *post hoc* tests to compare individual conditions (N = 3, ∗ for p < 0.05, ∗∗ for p < 0.01, ∗∗∗ for p < 0.001 and ∗∗∗∗ for p < 0.0001).

The STOX1-KO BeWo cell line (#5) was analyzed in triplicates by comparison with a wild-type (WT) BeWo, in the context of fusion, induced by a three-day treatment by FSK. We carried out transcriptomic analysis on this cell line using the ClariomD microarrays, and we exclusively extracted the annotated genes, resulting in a list of 47,488 transcripts. Among the deregulated genes, we could identify genes that were inversely affected by FSK in the KO compared to the WT cells. In this case, this tended to affect in particular non-coding RNAs, such as microRNA (miRNA) and circular RNAs (MIR4514, MIR4454, MIR520D, SPDYE8P, MIR3935, MIR509), as shown by some examples in Figure 1 for the genes framed in blue.

In the STOX1 KO, some genes were more upregulated by FSK than in the WT cells. These genes were frequent in pathways related to stress and differentiation. For instance, one of these genes was *ERVFRD1* which encodes for Syncytin2, the major endoretrovirus involved in trophoblast fusion (a terminal differentiation event of trophoblast cells), which could indicate that STOX1 modulates this function of cell fusion. This is substantiated for instance by the upregulation of *CYP19A1* (Aromatase), known to be increased in fusing trophoblast.^16^ In the present experiment, this gene was induced 14-fold in WT BeWo, but 86-fold in the STOX1 KO cells. The Syncytin 2 gene itself was induced 10.6-fold in the WT and 69.6-fold in the KO. Similarly, the CGB gene cluster on chromosome 19 was induced on average 19-fold in the WT and 29-fold in the KO, evidencing the fact that all these markers of trophoblast fusion are overreactive to cAMP signaling when STOX1 is absent.

**Figure 2:**
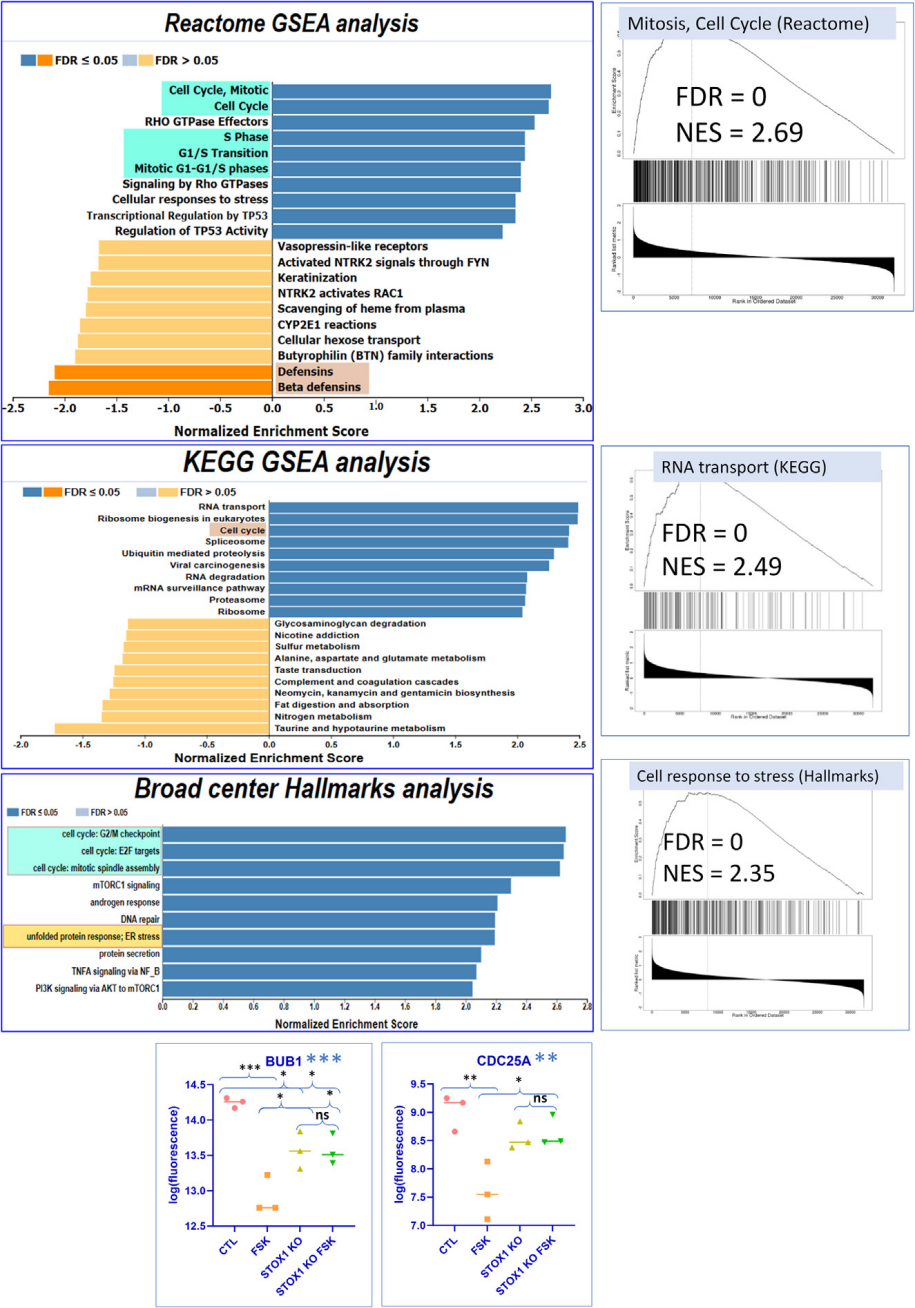
Transcriptomics analysis of STOX1 knockout in BeWo cells Gene set enrichment analysis (GSEA) carried out using WebGestalt, against three databases of pathways (Reactome, KEGG, and Hallmarks from the Broad Institute), to compare the effect of FSK in the context of WT BeWo or in STOX1 KO cell lines. In dark blue or dark orange are presented pathways with significant FDR (false discovery rate p < 0.05), composed of either overexpressed (blue) or downregulated (orange) genes. Striking pathways are boxed in color. Examples of GSEA curves are presented for Mitosis, Cell response to stress, and RNA transport. NES refers to normalized enrichment score (to be compared with 1 – no enrichment). In blue are presented two examples of cell-cycle genes, BUB1 and CDC25A, that are strongly downregulated by FSK in normal BeWo cells, and not modified anymore in the KO cells. These expression levels were analyzed by ANOVA followed by Tukey’s *post hoc* tests to compare individual conditions (N = 3, ∗ for p < 0.05, ∗∗ for p < 0.01, ∗∗∗ for p < 0.001; significant tests are shown, ns, is for not significant to emphasize the absence of FSK effect when STOX1 is KO for the two genes BUB1 and CDC25A).

Next, the transcriptome data were globally analyzed by gene set enrichment analysis (GSEA) using the WebGestalt platform against three general pathway databases, Hallmarks, Reactome, and Kyoto Encyclopedia of Genes and Genomes (KEGG). (Figure 2). The different analyses consistently revealed enrichment in cell-cycle genes in the KO compared to the WT cells under FSK treatment, as well as genes involved in stress response. *A priori*, it is expected that fusion (a terminal differentiation of the trophoblast) would be correlated with a halt in cell-cycle progression. With regards to cell fusion, we, therefore, got an apparently paradoxical observation: an enhanced increase of fusion markers such as Syncytin2 in the KO, together with an increased level of cell-cycle genes, while fusion, as a terminal differentiation, would rather be expected to be connected with a blockade of the cell cycle. The GSEA process applied was carried out by subtracting the induction level in the KO under FSK treatment minus the induction level in the WT. A positive value will thus originate from genes that negatively regulate the cell cycle in WT cells.

Indeed, as shown by the two examples given in Figure 2 for major genes of the cell cycle, such as *BUB1* and *CDC25A*, the effect of FSK was a strong downregulation (−2.8-fold and −3.1-fold, respectively) of the cell cycle in the WT while in the KO two things happened: the expression level of these genes was higher at the basal level in the KO (1.6-fold for *BUB1* and 1.4-fold for *CDC25A*), but the FSK treatment had no effect anymore. We also showed that under FSK treatment the transcripts of stress genes were increased in the KO, suggesting basal anomalies of these cells compared to the WT.

### Characterization of the structural and functional effect of STOX1 variants in the STOX1-KO BeWo cell line

We introduced the three mutations/variants located inside the open reading frame of STOX1 in the two aforementioned STOX1-KO cell lines followed by a selection of permanent transfectants with Geneticin G-418, following the protocol described by Ducat et al. and Rigourd et al.^2^^,^^3^ To compensate for possible specificities of isolated clones, we collected three independent clones per variant and first evaluated the level of expression of STOX1 by RT-qPCR, in two independent experiments carried out from two independent cultures, after two independent freezing/de-freezing cycles from liquid nitrogen (Figure S1). The two experiments yielded consistent results in term of relative expression between the cell lines, albeit the variability was high.

The 24 clones were then systematically analyzed by microarray analysis, while further analyses were carried out by live-cell imaging on the cell lines expressing the highest levels of WT or mutated versions of STOX1, which are labeled in red in Figure S1. The transcriptome analysis was carried out using the ClariomD tool (Thermo Fisher Scientific, Affymetrix), which allowed us to interrogate gene expression at the global gene, as well as at the exon level. The analysis was done after normalization by the 6 cell lines where the STOX1 WT version was overexpressed, considered as a baseline. Using this method, 2,300 transcripts were identified as significantly deregulated by ANOVA. A short list of the 40 most deregulated transcripts (20 up and 20 down) is presented in Table 1 for the three mutants, taken as a whole, and sorted according to increasing p value (ANOVA, 1 factor), and for transcripts varying in the same direction in all three mutants.

**Table 1:** List of the 40 most deregulated genes comparing each STOX1 mutant/variant to the WT

WT	Gene symbol	R364X	p value	Gene symbol	T188N	p value	Gene symbol	Y153H	p value
1	MT1X	13.24	2.93 10-2	MAMDC2	6.30	2.94 10-2	STS	4.81	2.46 10-2
1	MT2A	10.50	4.21 10-2	OR5P2	3.90	1.61 10-2	C1orf21	2.98	3.91 10-2
1	MT2P1	8.47	3.75 10-2	SFRP1	3.56	1.49 10-2	MIR4528	2.66	1.25 10-3
1	MT1B; MT1CP	6.25	2.54 10-2	RNU5D-2P	3.18	2.80 10-2	HIST1H1B	2.57	2.72 10-2
1	MT1A	5.99	4.38 10-2	UCA1	2.93	9.29 10-3	ANKRD6	2.53	3.57 10-2
1	MT1L	5.94	4.38 10-2	NPPB	2.71	6.26 10-3	RNA5SP335	2.47	4.14 10-2
1	MT1F	5.40	6.77 10-3	TMEM40	2.70	4.40 10-2	DDIT4	2.42	3.90 10-2
1	CSRP1	4.92	9.22 10-3	MRAS	2.48	6.65 10-3	UCP2	2.42	1.32 10-2
1	LGALS1	4.90	3.70 10-4	OR5P2	2.40	3.65 10-2	MYBL2	2.39	1.50 10-2
1	MT1DP	4.43	4.11 10-2	ID3	2.39	3.07 10-2	PTGES2	2.36	4.50 10-3
1	TWSG1	4.25	3.28 10-4	SCARNA6	2.28	1.28 10-2	FSCN1	2.35	2.85 10-2
1	YPEL2	4.08	6.93 10-4	SCARNA6	2.28	1.28 10-2	WARS	2.33	1.20 10-3
1	DDIT4	3.96	1.96 10-2	CH17-360D5.3	2.25	3.82 10-2	CSRP1	2.31	3.09 10-3
1	ITGA5	3.95	6.47 10-3	RNU6-455P	2.25	1.35 10-2	HIST2H4A; HIST2H4B	2.30	1.72 10-2
1	MT1G	3.69	3.75 10-2	KLRF2	2.23	2.97 10-2	RNU11	2.29	4.14 10-2
1	MT1XP1	3.50	2.55 10-2	SCARNA5	2.21	2.45 10-2	HIST1H3H	2.29	2.56 10-2
1	FAR2	3.42	2.75 10-2	SCARNA5	2.21	2.45 10-2	HIST1H2BL	2.28	3.69 10-3
1	ASS1	3.39	7.61 10-3	RNU5E-1	2.19	2.66 10-2	EPHX1	2.28	1.23 10-2
1	SASH1	3.38	2.59 10-2	MAATS1	2.18	1.00 10-3	SSRP1	2.26	1.23 10-2
1	RALBP1	3.20	2.37 10-6	SNORA80E	2.15	2.26 10-3	OGFRL1	2.24	3.69 10-2
1	AC007435.1	−2.44	5.18 10-4	CTSC	−2.25	3.63 10-2	LHFP	−1.94	3.16 10-2
1	CCL28	−2.44	4.70 10-2	FAM13A	−2.26	2.90 10-2	RRM2B	−1.95	4.58 10-2
1	AL136221.3	−2.46	1.04 10-2	MATN2	−2.28	2.70 10-2	CTD-2620I22.3	−1.97	2.09 10-2
1	FTX_3	−2.50	5.29 10-4	SNAI1	−2.29	4.03 10-2	LINC00632	−1.97	1.99 10-3
1	MIR520B	−2.51	1.57 10-3	HSD17B1	−2.30	4.43 10-2	AC007919.1	−1.98	1.36 10-2
1	DMKN	−2.53	2.15 10-2	DLX5	−2.32	2.80 10-2	H19_1	−2.00	4.82 10-2
1	MIR526B	−2.56	5.18 10-4	DNMT3L	−2.36	2.95 10-2	KB-68A7.2	−2.01	4.25 10-2
1	LINC01087	−2.57	1.30 10-3	PDE4DIP	−2.38	1.02 10-2	RP11-291I6.2	−2.03	4.60 10-2
1	MIR517A	−2.58	1.03 10-2	CDC42SE2	−2.39	1.91 10-2	MIR3189	−2.03	3.34 10-2
1	AL136221.2	−2.59	1.76 10-3	SYCP2	−2.49	6.20 10-3	AC078794.1	−2.05	3.68 10-3
1	MIR515-1; MIR515-2	−2.59	4.59 10-3	RP11-349F21.5	−2.50	9.74 10-3	AL844165.1	−2.05	1.35 10-2
1	MIR515-1; MIR515-2	−2.59	4.59 10-3	FAM101B	−2.58	3.29 10-2	RNU7-129P	−2.06	2.03 10-4
1	CTD-2314B22.1	−2.61	6.76 10-3	AC106736.1	−2.60	2.53 10-2	LNX1	−2.09	1.12 10-2
1	FTX	−2.66	1.96 10-4	MIR548AU	−2.77	1.00 10-2	ANKRD20A19P	−2.10	2.40 10-2
1	AC093865.1	−2.79	4.70 10-3	IL2RB	−2.97	2.41 10-2	SNORD123; SNHG18	−2.32	2.91 10-2
1	MIR17HG; MIR17; MIR18A; MIR19A; MIR19B1; MIR20A; MIR92A1	−2.85	4.59 10-3	TSPEAR-AS1	−3.00	1.75 10-2	RP11-20D14.6	−2.47	2.44 10-2
1	CTD-2240J17.3	−2.87	4.59 10-4	FNIP2	−3.05	2.31 10-3	MRGPRD	−2.57	1.99 10-2
1	MIR19B2	−2.95	4.59 10-5	FLT4	−3.11	3.34 10-2	P4HA1	−2.68	1.13 10-2
1	RP11-425M5.7	−3.02	4.59 10-6	RP11-20D14.6	−3.49	1.38 10-3	PRRG1	−4.17	1.12 10-2
1	RP11-20D14.6	−3.83	4.59 10-7	ANGPT4	−3.53	4.04 10-2	CDR1	−10.67	1.66 10-2

The analysis by *post hoc* Student’s t test revealed 2,850 differential genes with a statistical threshold of 0.05 with the R364X mutation compared to the WT, 2,013 with the T188N mutation, and 2,430 with the Y153H mutation. We used a GSEA approach against the Broad “Hallmarks” database to characterize the variations observed (Table 2).

**Table 2:** GSEA analysis of gene deregulations induced by mutated STOX1 versions relative to STOX1 WT

Gene set	Description	Size	Leading edge number	ES	NES	p value	**FDR**
**R364X vs. WT**							

HALLMARK_TGF_BETA_SIGNALING	TGF beta signaling	53	21	0.61696	2.0312	<2.2e-16	<2.2e-16
HALLMARK_UNFOLDED_PROTEIN_RESPONSE	unfolded protein response; ER stress	100	41	0.55794	2.0288	<2.2e-16	<2.2e-16
HALLMARK_HYPOXIA	response to hypoxia; HIF1A targets	192	62	0.52216	2.0284	<2.2e-16	<2.2e-16
HALLMARK_E2F_TARGETS	cell-cycle progression: E2F targets	184	86	0.52269	2.0104	<2.2e-16	<2.2e-16
HALLMARK_MITOTIC_SPINDLE	cell-cycle progression: mitotic spindle assembly	196	90	0.51832	2.0065	<2.2e-16	<2.2e-16
HALLMARK_EPITHELIAL_MESENCHYMAL_TRANSITION	epithelial mesenchymal transition	193	80	0.52288	1.9971	<2.2e-16	<2.2e-16
HALLMARK_P53_PATHWAY	p53 pathway	191	61	0.51505	1.9767	<2.2e-16	<2.2e-16
HALLMARK_OXIDATIVE_PHOSPHORYLATION	oxidative phosphorylation and citric acid cycle	176	76	0.51129	1.9577	<2.2e-16	<2.2e-16
HALLMARK_G2M_CHECKPOINT	cell-cycle progression: G2/M checkpoint	185	73	0.50359	1.9243	<2.2e-16	<2.2e-16
HALLMARK_PI3K_AKT_MTOR_SIGNALING	PI3K signaling via AKT to mTORC1	100	35	0.52515	1.8934	<2.2e-16	0.000084908

**T188N vs. WT**							

HALLMARK_E2F_TARGETS	cell-cycle progression: E2F targets	184	82	−0.5311	−2.3746	<2.2e-16	<2.2e-16
HALLMARK_G2M_CHECKPOINT	cell-cycle progression: G2/M checkpoint	185	76	−0.54644	−2.4446	<2.2e-16	<2.2e-16
HALLMARK_P53_PATHWAY	p53 pathway	191	69	0.41104	1.8829	<2.2e-16	0.00091613
HALLMARK_MYC_TARGETS_V1	MYC targets, variant 1	182	67	−0.42247	−1.8874	<2.2e-16	0.0015008
HALLMARK_MYC_TARGETS_V2	MYC targets, variant 2	53	26	−0.52491	−1.8886	<2.2e-16	0.001876
HALLMARK_ANDROGEN_RESPONSE	androgen response	93	27	−0.48583	−1.9574	<2.2e-16	0.0020011
HALLMARK_GLYCOLYSIS	glycolysis and gluconeogenesis	194	57	−0.39906	−1.7842	<2.2e-16	0.003752
HALLMARK_MTORC1_SIGNALING	mTORC1 signaling	194	55	−0.38388	−1.714	<2.2e-16	0.0050027
HALLMARK_CHOLESTEROL_HOMEOSTASIS	cholesterol homeostasis	70	19	−0.44207	−1.7188	<2.2e-16	0.005628
HALLMARK_APICAL_SURFACE	membrane proteins in the apical domain	43	16	−0.49299	−1.7206	0.003937	0.006432
HALLMARK_MITOTIC_SPINDLE	cell-cycle progression: mitotic spindle assembly	196	65	−0.36418	−1.6315	0.0019268	0.009305

**Y153H vs. WT**							

HALLMARK_E2F_TARGETS	cell-cycle progression: E2F targets	184	99	0.69341	2.7442	<2.2e-16	<2.2e-16
HALLMARK_G2M_CHECKPOINT	cell-cycle progression: G2/M checkpoint	185	95	0.68525	2.7003	<2.2e-16	<2.2e-16
HALLMARK_MYC_TARGETS_V2	MYC targets, variant 2	53	29	0.67208	2.2256	<2.2e-16	<2.2e-16
HALLMARK_MYC_TARGETS_V1	MYC targets, variant 1	182	97	0.54333	2.1254	<2.2e-16	<2.2e-16
HALLMARK_MITOTIC_SPINDLE	cell-cycle progression: mitotic spindle assembly	196	96	0.51369	2.0062	<2.2e-16	0.00017726
HALLMARK_OXIDATIVE_PHOSPHORYLATION	oxidative phosphorylation and citric acid cycle	176	94	0.51082	1.9982	<2.2e-16	0.00029543
HALLMARK_UNFOLDED_PROTEIN_RESPONSE	unfolded protein response; ER stress	100	40	0.49015	1.8111	<2.2e-16	0.00075969
HALLMARK_UV_RESPONSE_UP	UV response: upregulated genes	155	54	0.46519	1.7811	<2.2e-16	0.00077552
HALLMARK_ESTROGEN_RESPONSE_EARLY	early estrogen response	190	56	0.4228	1.657	<2.2e-16	0.0050224
HALLMARK_ESTROGEN_RESPONSE_LATE	late estrogen response	193	65	0.41184	1.6183	<2.2e-16	0.0063653

A striking observation was that, overall, the enriched gene ontologies (in this case “Hallmarks”) are substantially different from one mutant to the other, but, directly or not, E2F3-mediated cascades were affected; in Table 2, the normalized enrichment scores (NESs) were −2.37 for the T188N mutation, +2.74 for the Y153H variant, and +2.01 for the R364X mutation, with false discovery rate (FDR) p value <2.10^−16^, in the three cases.

**Figure 3:**
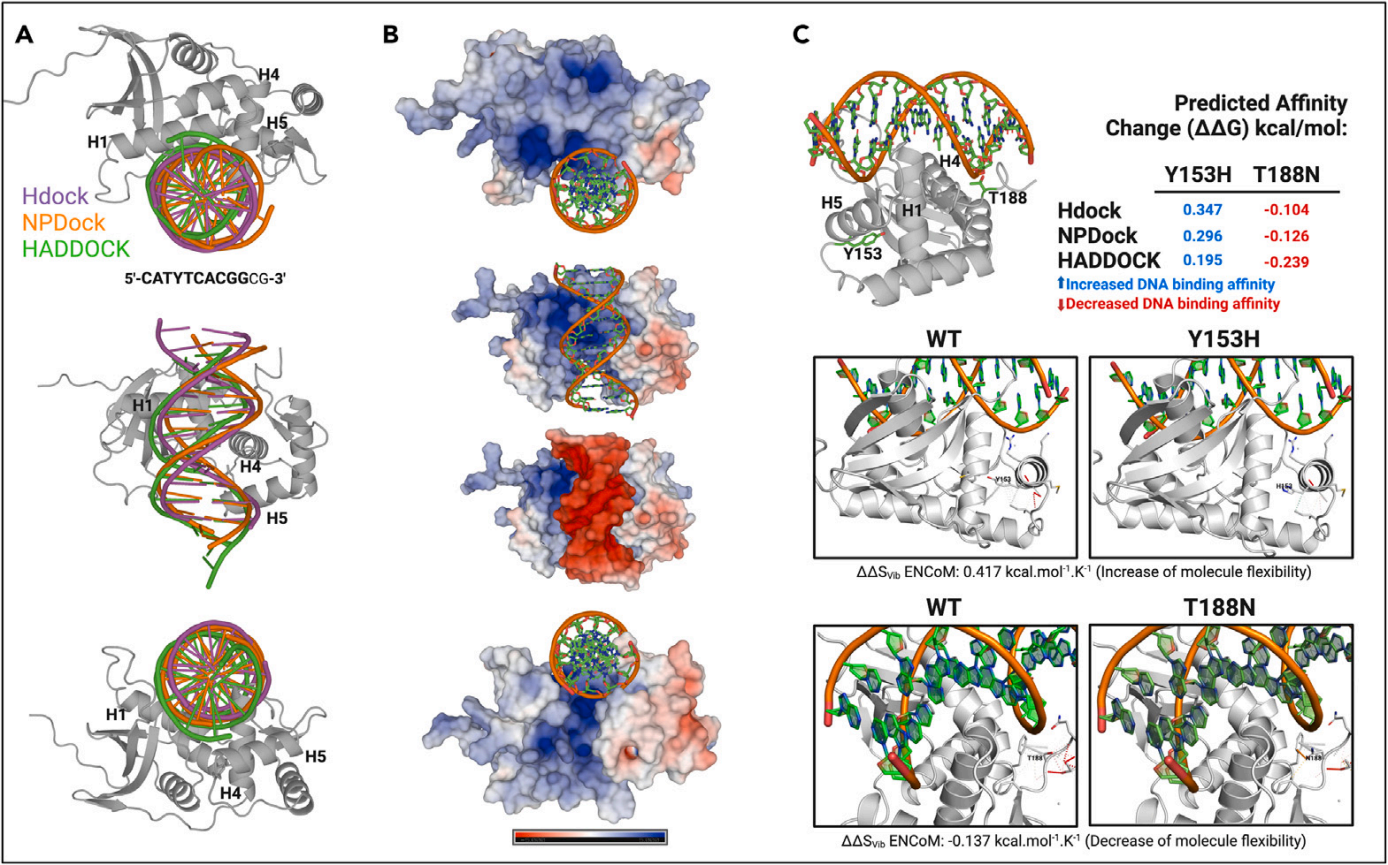
*In silico* analysis of the DNA-binding interface of STOX1 (A) Results of the blind docking of a B-DNA molecule (STOX1 binding site 5′-CATYTCACGGcg-3′) and the globular domain of STOX1 using three different tools: HDock, NPDock, and HADDOCK. The three tools showed consistent binding modes, mainly mediated by Helix 1, 4, and 5. (B) Electrostatic surface potentials of the protein-DNA binding interface. Residues are colored red and blue for negative and positive charges, respectively, and white color represents neutral residues. (C) The effects of mutations on protein-DNA interactions were calculated for T188N and Y153H. Using the three binding modes derived from each docking tool, the protein-DNA affinity change was consistently positive for Y153H and negative for the T188N variant, suggesting an increase and decrease in affinity, respectively. The bottom panel shows the structural effect of each mutation on the intermolecular interactions, showing weaker interactions for Y153H and stronger interactions for T188N. In fact, the Δ vibrational entropy energy between wild type and mutant for Y153H was predicted to increase protein flexibility (0.417 kcal mol^−1^.K^−1^) whereas for T188N was predicted to decrease protein flexibility (−0.137 kcal mol^−1^.K^−1^).

To further characterize the structural impact of the T188N and Y153H variants on STOX1, we used *in silico* analysis (see STAR Methods) to predict the DNA-binding interface of STOX1 to a previously described DNA motif which is the STOX1 binding site, STRE1, CATYTCACGG.^2^ The three independent blind docking analyses consistently showed the same protein-DNA interface (Figure 3A). Additionally, further analysis confirmed that this interface is positively charged, which is critical to driving the electrostatic interaction between the surface of the protein and the negatively charged DNA molecule (Figure 3B). More importantly, *in silico* predictions using the three consistent binding modes derived from docking showed an increased binding affinity for the Y153H variant and a decreased binding affinity for the T188N mutation (Figure 3C). This observation also fits remarkably well with the fact that no significant enrichment in binding sites was detected while comparing T188N with WT transcriptomic data, while there is a plethora of significant enrichments with Y153H, especially connected with E2F targets (Figure S3).

**Figure 4:**
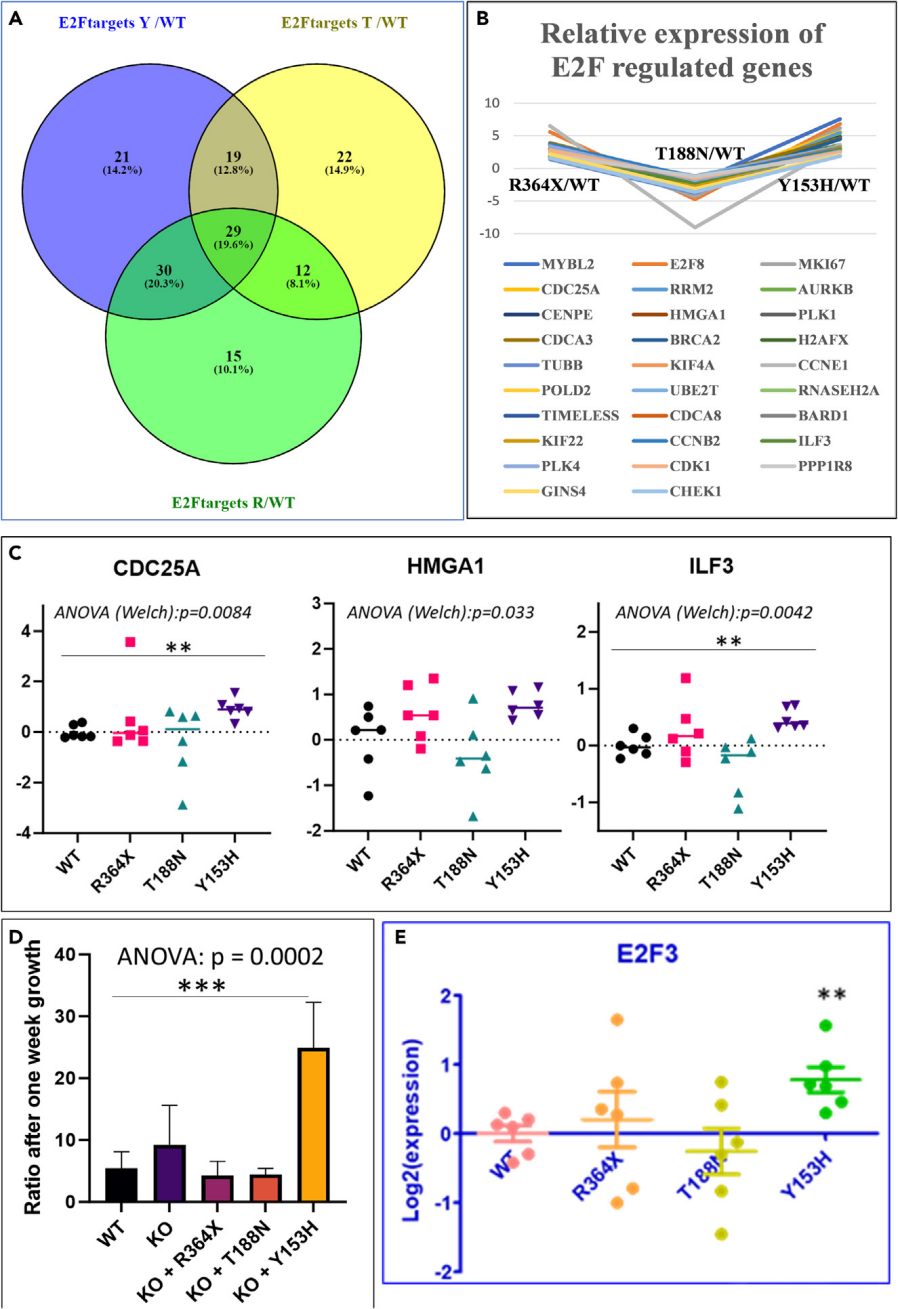
Cell cycle mediated by E2F signaling was found as a pathway systematically modified through overexpression of any of the three variants analyzed The leading edge of this cascade presented with 29 genes modified in common (A), comparing Y153H versus WT in blue, T188N versus WT in yellow, and R364X versus WT in green. (B) Comparison of the deregulation of common genes from the leading edge, showing a deregulation that is similar in the R364X and Y153H mutation, and opposite following the T188N overexpression (ordinates are the level of fluorescence relative to the WT overexpressing cells). Examples of gene deregulation are given in (C), analyzed by ANOVA followed by *post hoc* Dunnett test against the WT, mainly driven by the difference between Y153H and WT. (D) The cell growth was also significantly altered showing an accelerated growth of the cell harboring the Y153H variants, while growth appeared inhibited by the T188N and R364X mutations, without reaching *post hoc* significance. Consistently, there is a significant induction of one member of the E2F family, E2F3 (E), a transcription factor harboring a major importance in trophoblast biology, significant specifically in the cells overexpressing the Y153H variant (∗∗ for p < 0.01, ∗∗∗ for p < 0.001).

There were 29 genes in common for the three mutants analyzed that are targets of the E2F transcription factors, as shown in Figure 4A (*AURKB*, *BARD1*, *BRCA2*, *CCNB2*, *CCNE1*, *CDC25A*, *CDCA3*, *CDCA8*, *CDK1*, *CENPE*, *CHEK1*, *E2F8*, *GINS4*, *H2 AFX*, *HMGA1*, *ILF3*, *KIF22*, *KIF4A*, *MKI67*, *MYBL2*, *PLK1*, *PLK4*, *POLD2*, *PPP1R8*, *RNASEH2A*, *RRM2*, *TIMELESS*, *TUBB*, *UBE2T*). All these genes are downregulated by the T188N mutation and upregulated by the Y153H mutation and the R364X mutation (Figure 4B). Besides these global effects, and despite the relative heterogeneity of the expression levels in the different cell lines, several genes are significantly deregulated at the individual level as exemplified in Figure 4C. We explored cell growth after one week in culture and found that there was a significant variation in the growth rate between the different cell lines, this difference being mainly driven by the Y153H mutation, as revealed by the *post hoc* Dunnett test (Figure 4D). By contrast, the R364X mutation was unable to promote accelerated growth, despite a tendency to modulate gene expression in genes connected to proliferation in a way similar to the Y153H mutation. As shown in the examples presented in Figure 4C, this trend to increased expression of cell-cycle genes by the R364X mutant is however less marked than that by the Y153H variant for the genes presented as examples, and the NES, for instance for the Hallmark “E2F targets” (connected to proliferation), is only of 2.01 in R364X versus 2.74 in Y153H, corresponding to a much more striking effect of this latest mutation on proliferation. The direct effects of the mutants on the proliferation of the cells were validated through live-cell imaging (Figure S2). In this case it was possible to show that the R364X and T188N mutants had indeed an effect in slowing down the proliferation.

This effect on cell proliferation was examined to see whether it was a direct effect of the STOX1 variants added or not. To analyze this, we ran a GSEA against the Transcription Factor Binding Site (TFBS) database, and we found a very significant enrichment of genes containing E2F-binding sites in the promoters of the genes activated by the Y153H variant, especially E2F3 (Figure S3). We further analyzed the relative expression of this transcription factor in the Y153H versus the WT cells and found a significantly increased abundance of the E2F3 transcript, specifically in this mutant (Figure 4E). Therefore, the E2F3 action is directly under the control of the Y153H mutant, while the increased expression of E2F3 target genes in the R364X mutant is indirect and could be an additional element (together with the activation of the transforming growth factor β [TGF-β] cascade) to explain why in these cells, despite the enrichment, there is no specific increase in proliferation following overexpression of this STOX1 mutant.

Archetypal targets of the R364X mutant compared to the WT cells are the metallothionein complex of genes on chromosome 16 (from 2- to 5.76-fold), LGALS1 (x4.48-fold), or HTRA4 (x22.81), genes known to be connected with preeclampsia. In the case of T188N, one of the major genes modified is the long non-coding RNA *UCA1* (upregulated 4.06-fold and known as a major actor of trophoblast proliferation, and function).^17^^,^^18^^,^^19^

### Live-cell analysis of STOX1 mutants in growth, fusion, and oxidative stress management

Trophoblast cell fusion is classically followed experimentally by analyzing the fluorescence labeling of a marker of the cell membrane together with fluorescent labeling of the nucleus. Here we opted for a fluorescence labeling protocol recently set up,^20^ using DI-8-ANEPPS, a labeler of the cell membranes, that we could first apply to BeWo cells. We then attempted to analyze the fluorescence automatically using the IncuCyte live-cell imaging platform (Sartorius).

#### Growth measurement

**Figure 5:**
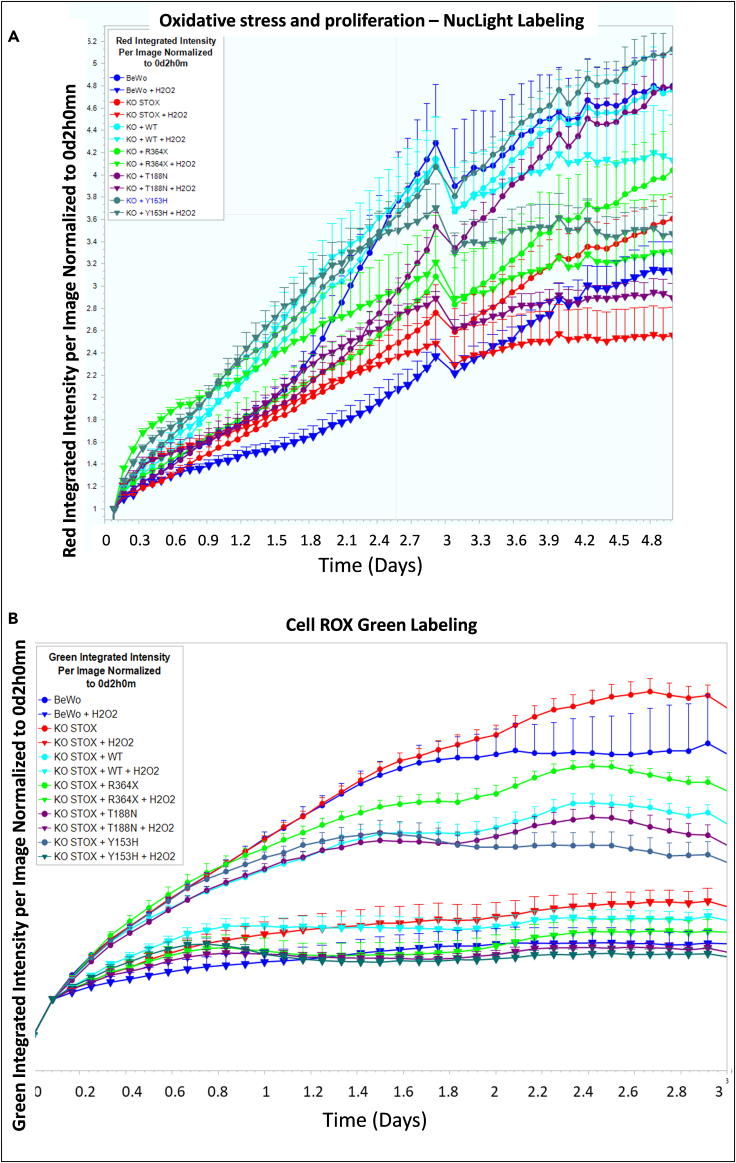
Live-cell analysis of oxidative stress effects (A) An example of live-cell imaging for proliferation in the presence or absence of oxidative stress. Circles represent non-treated cells, while inverted triangles correspond to cells treated with 100 μM H_2_O_2_. (B) Measure of superoxide ions was performed by fluorescent labeling of the cells with CellROX green (Thermo Fisher Scientific), without (circles) or with (inverted triangles) H_2_O_2_ treatment. To note, the drop at day 3 was due to an opening of the machine for another contemporaneous experiment. In both images, the error bars are provided by the Incucyte, resulting from two to four replicates (each replicate being constituted from four quadrants).

**Figure 6:**
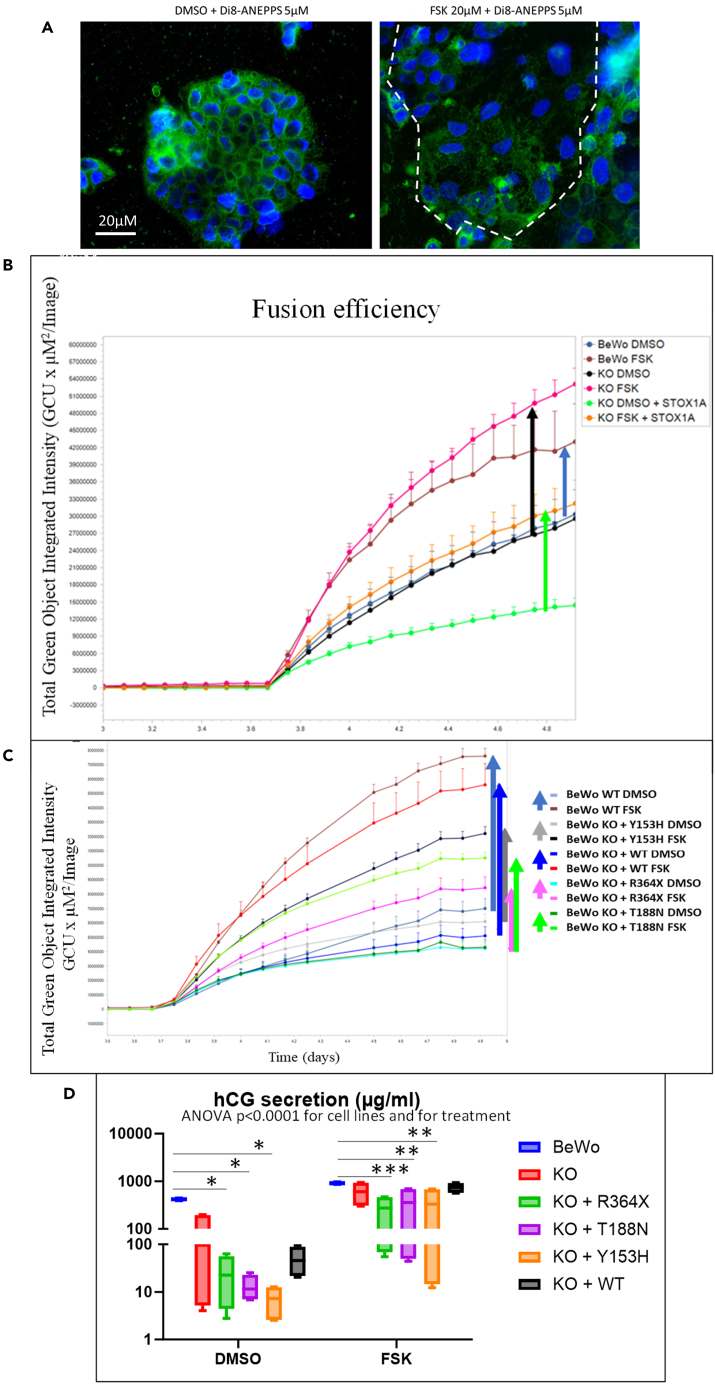
Analysis of trophoblast cell fusion The analysis was achieved by classical immunofluorescence using the Di8-ANEPPS fluorophore (A), and live-cell imaging in the Incucyte device. Forskolin (FSK, 20 μM) was added for three to four days, and the fluorophore was added ∼36 h before the end of the experiment. In (B) is presented the increase of fluorescence detected in each well image (divided in four compartments). The increase in green fluorescence is marked by the arrows for BeWo WT cells (blue), STOX1 KO cells (black), and BeWo cells where only STOX1A is overexpressed (green). The same type of analysis in an independent experiment comparing the effects of the different variants. The length of the arrow refers to the efficacy of cell fusion, showing the following order: WT > KO + WT >> KO + T > KO + Y >> KO + R (C). (D) Measure of the secreted hCG in the culture medium by ELISA with and without FSK treatment (tested by 2-ways ANOVA followed by Dunnette t test using BeWo cells as a reference, ∗: p < 0.05, ∗∗p < 0.01, ∗∗∗p < 0.001). In images (B) and (C), the error bars are provided by the Incucyte, resulting from two to four replicates (each replicate being constituted from four quadrants).

**Figure 7:**
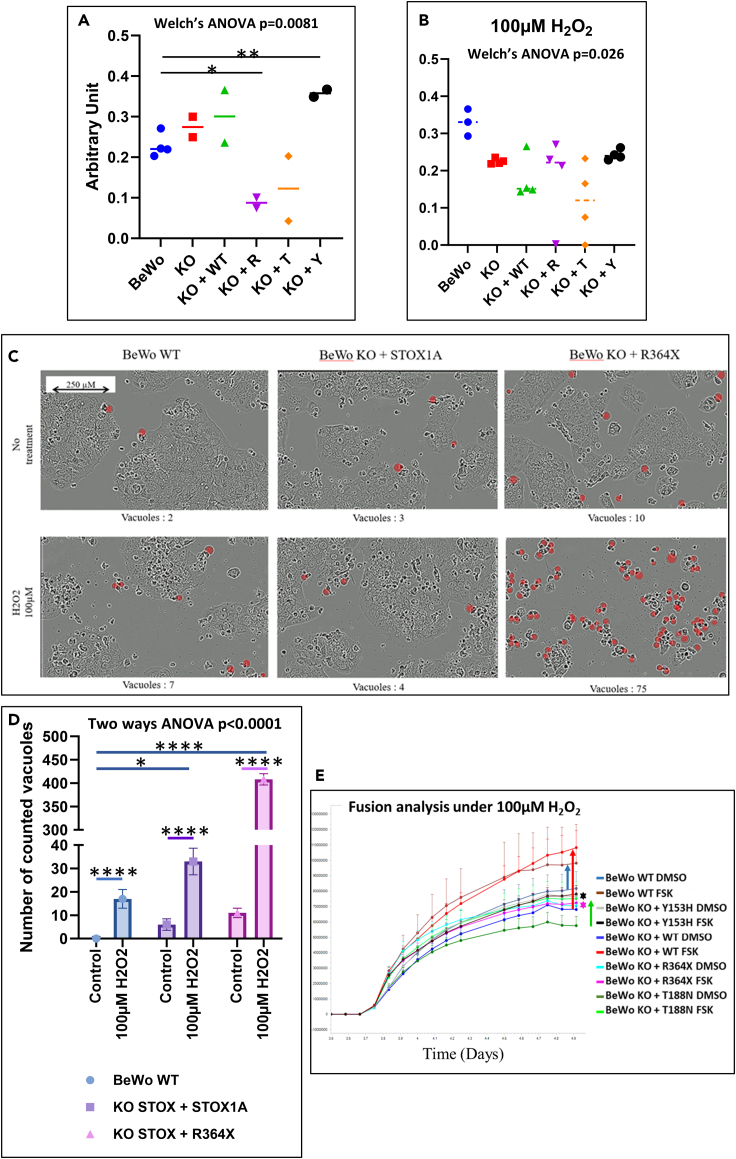
Management of oxidative stress by STOX1 variants Survival/proliferation tests were carried out using the CCK8 approach, without added oxidative stress (A), or following treatment with 100 μM H_2_O_2_ (B). A bright-field vision of BeWo WT, BeWo KO + STOX1A, and BeWo KO + R364X STOX1A mutant (C). The vacuoles that are a mark of cell death were counted manually from two independent experiments, represented numerically in (D) for the complete field. The fusion was analyzed in the mutants with oxidative stress treatment, showing a complete inhibition of fusion in the Y153H mutant and in the R364X mutant (black and pink stars), and fusion maintained at least at a certain level for the T188N (green arrow), the KO + WT (red arrow), and the standard BeWo cells (blue arrow) (E). In image (E), the error bars are provided by the Incucyte, resulting from two to four replicates (each replicate being constituted from four quadrants).

NucLight was used to quantify growth in the cell lines analyzed in the presence or absence of oxidative stress (100 μM H_2_O_2_ treatment, Figure 5A). The concentration was defined experimentally and following previous literature (Figure 6). Without oxidative stress, the growth was similar for BeWo cells and the STOX1 KO cells where STOX1A was added, either WT or the T188N, while the Y153H variant tends to enhance growth (see also Figure 7A). By contrast, the growth was altered when the R364X mutant was added or in the KO cells. In the presence of oxidative stress, the growth was blunted in all cell lines with a relative protection when the STOX1A expression was restored (KO + WT).

**Figure 8:**
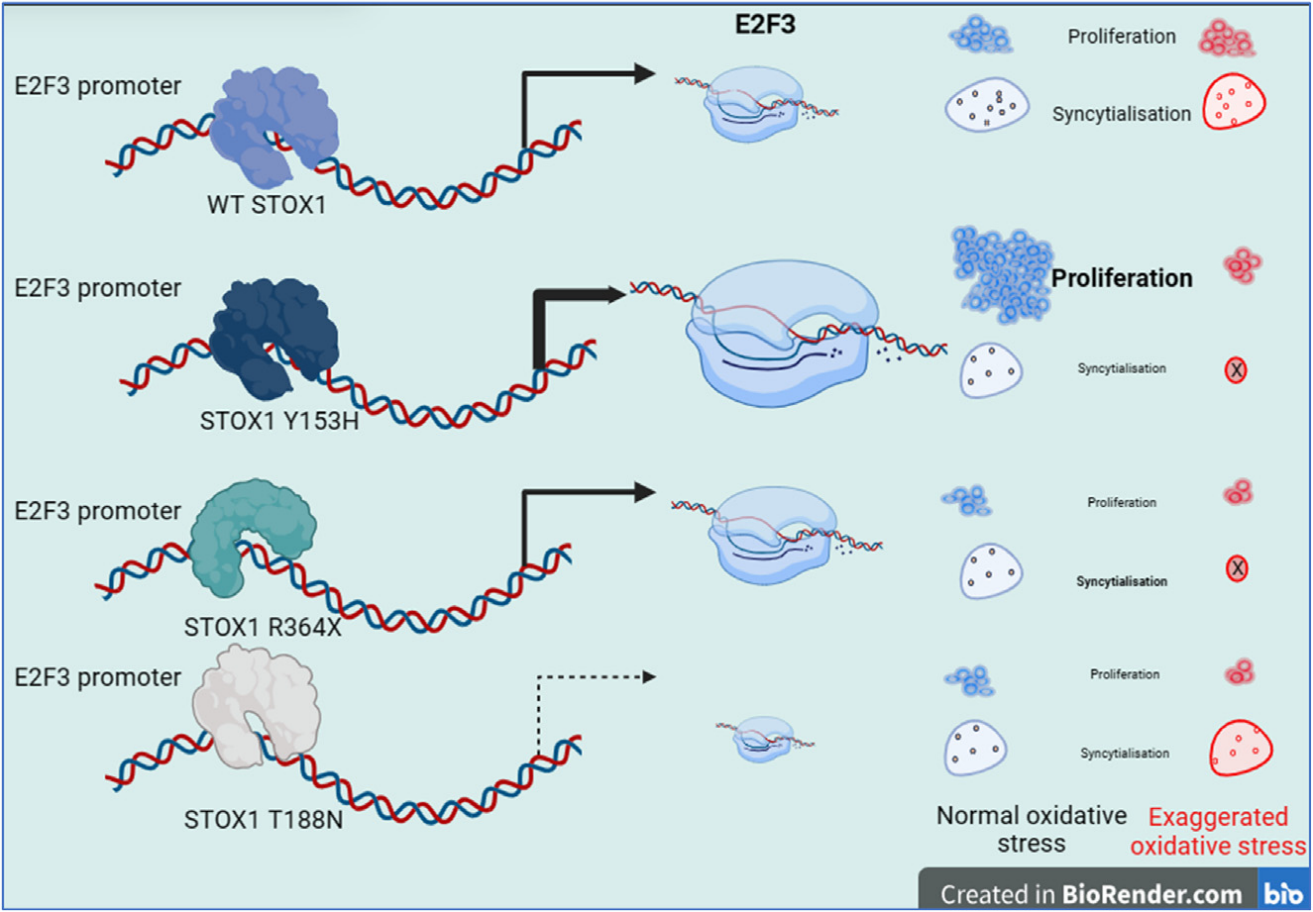
A summary of the major findings of the study The E2F3 transcription factor appears as a major transcriptional target for STOX1, and especially the Y153H variant leads to i) an increased flexibility of the binding site, ii) an increased affinity to the STOX1-responsive element 1 (SRE1), and iii) a higher expression of this factor with activation of its direct targets, whereas there are opposite effects of the T188N variant. This results in increased proliferation of cells expressing STOX1 Y153H, while FSK-induced syncytialization is rather reduced in all the mutants, consistently with a decrease production/secretion of hCG. In response to high levels of oxidative stress, an inevitable companion at some stages of placental development, the proliferation is slowed down in the cells with the variants, and syncytialization is even totally abolished with the cells expressing the Y153H and the R364X variants. These observations provide possible STOX1-dependent pathways underlying placental disease.

#### Superoxide ions measurements

One of the most important molecules leading to oxidative stress is the superoxide ion. It can be readily measured by fluorescence for instance using the CellROX fluorophore (Figure 5B). At the basal level, the level of superoxide increased during the culture of the BeWo cells, but more in the STOX1 KO cells, and less when the STOX1 WT, T188N, or Y153H expression was restored. The R364X mutant cells showed an intermediate behavior (Figure 5B).

#### Fusion

In the first series of experiments we compared the level of fusion in WT BeWo cells, STOX1 KO cells, and the KO where STOX1A was overexpressed by FSK treatment. The FSK was added one day after plating and the Di-8 at 3.7 days for a total duration of 5 days. In all the following independent experiments (>10), we observed that the green fluorescence signal was systematically increased by the FSK treatment (Figure 6B), possibly due to an entry inside the cells and labeling all the membranes. One striking observation was the fact that in the KO the labeling is almost twice as high as in the WT cells. In the KO where STOX1A is overexpressed, the intensity of fusion (labeling being considered as a proxy of this mechanism) was also increased more strongly than in the WT cells (compare the length of the black arrow with the length of the blue arrow [Figure 6B]). When STOX1A alone was added to the KO cells, then the fusion was at least as high as in the KO (green arrow in Figure 6B). In the following experiment (Figure 6C) we evaluated the variant Y153H, and the mutations R364X and T188N, in terms of fusion capability. Systematically, we showed that the three variants had a decreased capability of fusion compared to the added WT variant or to the WT BeWo cells in this experiment, which have more or less the same capability to fuse. The Y153H and T188N variants have a decreased capability to fuse compared to these two cell lines, but the most striking negative effect was triggered by the overexpression of the R364X variant (pink arrow compared to blue arrow). To measure the potential physiological effects of the different variants, we analyzed the secretion of β hCG in the culture medium of the cell lines by ELISA, since the production and synthesis of this hormone is one of the earlier marks of trophoblast fusion. Without FSK treatment the basal level of hCG was considerably reduced in KO cells and in all the derived cell lines, with a drastic additional reduction when any mutant version of STOX1 was overexpressed, especially the Y153H variant. FSK treatment increased the hCG production in all the situations (Figure 6D) but was not restored at the level observed in BeWo cells except in the KO and in the KO where the WT STOX1 was overexpressed.

#### Management of oxidative stress

Another pivotal aspect of STOX1 action is its capability of managing oxidative stress products as shown in a series of previous publications.^2^^,^^15^^,^^21^ We thus explored the effect of the addition of the different STOX1 variants upon this, using a treatment with hydrogen peroxide, H_2_O_2_; we first tested the dose response of the cells with 0, 5, 50, and 100 μM of H_2_O_2_ and showed that the WT BeWo cells support easily the highest concentration, in conformity with the published literature. Adding H_2_O_2_ reduced systematically the superoxide ion level in all the cell lines (Figure 5B). To examine the viability of the cells under this condition of oxidative stress, we performed Cell Counting Kit-8 (CCK8) viability tests and could observe that, in this case, there was a significant negative effect of the R364X mutant overexpression and a significant positive effect of the Y153H variant. There was a similar trend for the T188N mutant and the R364X mutant (Figure 7A). This was consistent with the observations presented in Figure 4D. When oxidative stress was applied (100 μM H_2_O_2_), the profile tended to show a decreased survival in all the mutants derived from BeWo cells (Figure 7B). Despite this trend, there was no inter-group significant variation. To complete the analysis, we moved to analysis of the cells by live imaging. This way, we demonstrated in particular a very strong sensitivity to oxidative stress in the R364X-overexpressing cells. We could see a massive increase of vacuolized cells in these conditions, leading to cell death, in conditions that were not obviously different from control conditions (without oxidative stress, Figures 7C and 7D). This substantiates the idea that STOX1 is a major player in regulating oxidative stress. The non-equivalence between WT BeWo (that express STOX1 as a proportion of STOX1A and STOX1B) and BeWo STOX1 KO where STOX1A alone is overexpressed is illustrated in Figures 7C and 7D, with a lessened survival and a relatively important increase of vacuolized cells. Assessing this quantitatively indicated a drastic deleterious effect of the R364X mutant when cells are exposed to oxidative stress (Figure 7D).

We also tested the impact of the oxidative stress in the various cell lines on the fusion process mediated by FSK treatment (Figure 7E). The BeWo WT and the KO with the overexpression of STOX1A were still able to fuse efficiently (blue and red arrows, respectively). By contrast, in the Y153H and R364X variant cells (black and pink stars), the fusion process was abolished (Figure 7E) while the T188N mutant appeared relatively resistant and able to fuse in oxidative stress conditions (green arrow). Interestingly, the degree of fusion in this stressful condition was apparently enhanced in the KO with overexpression of STOX1A (red arrow), which is consistent with a role of STOX1A in countering oxidative stress (but increasing nitrosative stress), in cell models and in the placenta,.^21^

## Discussion

Certain variants of the STOX1 transcription factor have been linked to pregnancy diseases, particularly hypertensive disorders of pregnancy. One of the most notable variants, Y153H, was first identified in a study that established the connection between STOX1 and preeclampsia.^1^ Subsequent investigation into this variant revealed that it affects trophoblast invasion by upregulating the alpha-T-catenin^6^ and decreasing the secretion of specific cytokines, including interleukin (IL) 6, IL8, CXCL16, and TRAIL. These changes have an impact on endothelial chemokine expression, angiogenesis, uNK, and monocyte migration.^8^ This variant is frequent (∼30% in most populations) and contrasts with two rare mutations found in HELLP syndrome (a hepatic complication of preeclampsia), T188N and R364X, identified following an exome sequencing (exome-seq) analysis of Colombian patients. Finally, a recent promoter variant of STOX1 (−922T>C) was associated with an increased risk of preeclampsia.^22^ In the present study, we analyzed the structural and physiological impact of mutations/variants on the open reading frame of STOX1 in the BeWo trophoblast cell model. We introduced these variants in an STOX1-KO background and evaluated their functional effects following transcriptional characterization of the invalidation.

One striking observation of the physiology of the BeWo STOX1 KO cells that we established in the present study is the overall trend that gene markers of trophoblast fusion (such as Syncytin 2, *ERVFRD1*) are rather more induced than in the WT cells, with an apparent better efficiency of fusion than in the WT cells. This may mean that the absence of STOX1 would influence negatively the cell cycle, promoting instead cell fusion under FSK treatment, this observation being vindicated by improved FSK-mediated induction of fusion genes (such as CGB and ERVFRD1). This is to be compared with the observation that the STOX1A isoform favors fusion while the STOX1B isoform prevents it.^2^ The enhancement of fusion and its consequences in the KO hence suggests that, in the normal situation, STOX1B plays a major role in quieting trophoblast fusion, while switching to an enhanced expression of STOX1A would play a part in promoting fusion. Therefore, removing all the STOX1 isoforms from the picture (as done by the gene invalidation) tends to tilt the balance toward fusion. These observations are also consistent with the fact presented here that the R364X mutant generates an isoform closer to STOX1B (truncated in the transactivating domain of STOX1A at 364 amino acids) than STOX1A (989 amino acids) in achieving its biological function, STOX1B (229 amino acids) being reported as countering fusion in the BeWo cell model.^2^ A summary of the results of the study, including the effects of oxidative stress are summarized in Figure 8.

When stable cell lines overexpressing the three mutants were obtained, we observed overall that each of them had widely different transcriptomic effects, with mostly different targets. Among the paradigmatic genes modified connected to placental disorders, we can mention the long non-coding RNA *UCA1*, upregulated more than 4-fold by the T188N mutant compared to the WT STOX1A. UCA1 is induced in the preeclamptic placenta^23^ and was shown to be connected to trophoblast cell migration,^19^ cell invasion and proliferation,^18^ trophoblast fusion,^17^ and the ability to trigger endothelial injuries through UCA1-loaded trophoblast exosomes.^24^ In the genes specifically induced by the R364X mutant, it is noticeable that the metallothionein gene cluster is upregulated. It has been recently shown that SNPs in these genes can be used as a risk signature of hypertensive disorders of pregnancy.^25^ In addition, the variant induces a systematic downregulation of a cluster of miRNAs located on chromosome 19, including MIR-515, -517a, -520e, -521, -520c, -526b, -520a, -518, etc. This cluster of miRNAs (cluster C19-C) is specific to the human placenta and plays a pivotal role in the function of this organ.^26^^,^^27^^,^^28^ Also, *LGALS1* is deregulated by the variant, while the invalidation of this gene triggers preeclampsia-like symptoms in mice.^29^

The effect of Y153H is more global, without a specific “preeclamptic gene” evidently modified at first sight, while at the ontology levels, as well as at the TFBS level, we show for the first time that the effect of this variant is, at least in part, mediated by a direct action on the E2F3 transcription factor, in addition to previously described pathways influenced by this mutation, such as the PI3 kinase or the NODAL signaling cascades.^6^^,^^8^^,^^30^^,^^31^ While the enrichment in E2F3 deregulated targets was milder in the R364X (positively) and with the T188N mutation (negatively), the E2F transcription factor targets were apparently systematically modified by STOX1 mutations, and this may have opposite consequences upon the cellular cycle, depending on the variants T188N and Y153H present in the cell. Phenotypically speaking, the R364X has no obvious improved proliferation, despite this positive enrichment of E2F3-regulated genes. It is hence possible that some alterations linked to the overexpression of R364X lead to compensatory effects mitigating the actual pro-proliferative effect. Indeed, the first enriched hallmark in the R364X cells is “TGF-β signaling,” a cascade abundantly documented as a negative regulator of cell proliferation and cell growth.^32^ Thus, in this cell line, there is together activation of genes of the cell cycle and activation of genes acting toward differentiation (and thus rather opposed to proliferation and growth). The physiological impact of the mutation in the R364X cell line eventually appears tilted toward a limitation of proliferation (Figure 8). A recent atlas of the human placenta at early terms incidentally pinpointed E2F3 as a major transcription factor of placental development, specifically of cytotrophoblast cell columns of the extravillous cytotrophoblast.^33^ This last study identified 27 transcription factors important for placental physiology with their cell-type specificity. Our results strengthen the observation and propels E2F3 in the position of an absolutely essential transcription factor in the normal dynamics of cell types in the human placenta, promoting proliferation against differentiation.

The frequency of the three STOX1 variants identified in the human population is extremely different; Y153H (corresponding to the rs1341667 variant) reaches a frequency of ∼36%–38% in Caucasians and South Asian populations, 9% in East Asian populations, and up to 62% in African populations (data from the 1000Genomes_30x dataset (https://www.ncbi.nlm.nih.gov/snp/rs1341667#frequency_tab). This might explain the incomplete penetrance and the role of other genes like NODAL, suggesting either digenic or oligogenic inheritance, or the role of STOX1 as a genetic modifier. The high frequency of the variant Y153H in African populations is consistent (certainly without underpinning a full causality) with the higher prevalence of preeclampsia in sub-Saharan African population. By contrast, the T188N and R364X mutations were found in four and one HELLP patients of Colombian origin (out of 79 patients and 176 ethnically similar controls), which suggest a minor allele frequency of less than 0.008 and 0.002 for T188N and R364X, respectively.^7^ The rarity and predicted deleteriousness of these mutations suggest that they have an important effect on placental function and are much less tolerable than the Y153H variant. It is even possible that, in certain environmental conditions, Y153H could be advantageous and positively selected. This may be a reason for actively maintaining a large proportion of this polymorphism despite its deleterious effect in terms of the risk of abnormal placental function and preeclampsia. These possibilities of balanced selection have been evoked previously.^34^

In our cell models we also observed unexpectedly that H_2_O_2_ treatment reduces superoxide ions, which are themselves used by SODs to generate H_2_O_2_. This unexpected observation was nevertheless consistent with the idea that superoxide dismutase gene expression is induced by H_2_O_2_ treatment.^35^ At the post-transcriptional level, it has also been shown that H_2_O_2_ is able to promote the action of SOD1 on specific substrates.^36^ Given the extremely high activity of this enzyme this could be consistent with the collapse of the superoxide ions concentrations when H_2_O_2_ is added to the cells.

Our work as presented here is a thorough analysis of STOX1 mutation effects. It is important to acknowledge certain limitations in this study. Firstly, the data collected were solely from trophoblast cell lines. Additionally, it should be noted that women with the T188N and R364X mutation are heterozygous, whereas in the cell only the mutated isoform is present. Nevertheless, our data provide insights into the ways STOX1 variation heavily modifies gene expression regulation in trophoblast cells.

Preeclampsia has a genetic component estimated at 50%.^37^ In familial cases, some variants will have a prominent effect such as the Y153H variant of STOX1 initially described in Dutch families.^1^ The question of the generality of the involvement of this gene in non-familial cases is less obvious; here we dissect the mode of action of three variants/mutations of STOX1, and a major observation is that each variant has different effects on trophoblast biology through the activation of different pathways perturbing trophoblast function. The activation of E2F3 seems to be a general track by which abnormal placental function (either acceleration of the differentiation process, or slowing down this process) can be triggered. This transcription factor could deserve a specific research focus for a better understanding of placental disease. The identification of a very frequent variant of STOX1 as having a strong molecular effect on trophoblast physiology may come as a surprise; if its effect is unambiguously negative, it should be counter-selected, suggesting that evolutionary fitness could be enhanced by this variant in specific environmental or genetic contexts.

In this paper, we elucidate, at least partly, the activity of STOX1 variants affecting the DBD. When the variant does not affect the DBD, such as in the R364X mutant, the effect could rather be mediated by physical interactions with protein partners. To progress mechanistically in the evaluation of such variants will necessitate other approaches that will be the topic of further research.

### Limitations of the study

One of the major limitations of this study is the use of cell models to demonstrate the effects of STOX1 variants. Nevertheless, numerous publications use BeWo for emulating trophoblast cells, which will make it possible to compare our results with other datasets. One prolongation of the project would be to introduce the mutations/variants in mice, but, to systematically screen the effects of the mutation, cells are extremely convenient and yield sound information about the mechanistical effects of the mutations.

## STAR★Methods

### Key resources table

**Table:** 

REAGENT or RESOURCE	SOURCE	IDENTIFIER
**Antibodies**		

anti-STOX1	Merck	PA5-65193

**Bacterial and virus strains**		

XL10-Gold ultracompetent cells	From Agilent Quickchange XL kit	#200521

**Chemicals, peptides, and recombinant proteins**		

Geneticin	Thermofisher Scientific	10131035
CellROX™ Green	Thermofisher Scientific	C10444
Nuclight Rapid Red Dye	Sartorius	
Forskolin	Sigma	F6886
Di-8-ANEPPS	Thermofisher Scientific	D3167

**Critical commercial assays**		

QuickChange II XL kit	Agilent, Santa Clara, CA	#200521
hCG-beta ELISA kit	Thermofisher Scientific	# EH235RB
CCK-8 kit	Merck	96992

**Deposited data**		

Microarray ClariomD Human BeWo KO vs. WT with or without forskolin	Deposited EMBL-EBI	E-MTAB-13352
Microarray ClariomD Human BeWo KO + WT or + R364X mutant, T188N mutant or Y153H mutant	Deposited EMBL-EBI	E-MTAB-13357

**Experimental models: Cell lines**		

BeWo cell line	ATCC	CCL-98
BeWo cell lines where STOX1v is invalidated	This study	KO#5
BeWo cell lines where STOX1v is invalidated	This study	KO#21
BeWo cell lines where STOX1v is invalidated with overexpression of STOX1 WT	This study	KO + WT
BeWo cell lines where STOX1v is invalidated with overexpression of STOX1 R364X	This study	KO + R
BeWo cell lines where STOX1v is invalidated with overexpression of STOX1 T188N	This study	KO + T
BeWo cell lines where STOX1v is invalidated with overexpression of STOX1 Y153H	This study	KO + Y

**Oligonucleotides**		

STOX1 expression forward	Eurogentec	CGGTGGGTGATGTCTTTCCA
STOX1 expression reverse	Eurogentec	CACAGCAAAGAACTTCACCCA

**Recombinant DNA**		

Plasmid encompassing a 6-flag mutant STOX1 with R364X	This study	pcDNA3.1 R364X
Plasmid encompassing a 6-flag mutant STOX1 with T188N	This study	pcDNA3.1 T188N
Plasmid encompassing a 6-flag mutant STOX1 with Y153H	This study	pcDNA3.1 Y153H

**Software and algorithms**		

NPDock	Tuszynska et al., Nucleic Acids Research, 2015 https://doi.org/10.1093/nar/gkv493^38^	https://genesilico.pl/NPDock/
Alphafold	Varadi et al., Nucleic Acids Research, 2022 https://doi.org/10.1093/nar/gkab1061^39^	https://alphafold.ebi.ac.uk/entry/Q6ZVD7/
HDOCK	Yan et al., Nucleic Acids Research, 2017 https://doi.org/10.1093/nar/gkx407^40^	http://hdock.phys.hust.edu.cn/
HADDOCK 2.4	De Vries et al., Nature Protocols, 2010 https://doi.org/10.1038/nprot.2010.32^41^	https://wenmr.science.uu.nl/haddock2.4/
PyMOL 2.5	Distributed by Schrödinger (Schrödinger - Physics-based Software Platform for Molecular Discovery & Design (schrodinger.com))	https://pymol.org/2/
mCSM-NA	Pires and Ascher, Nucleic Acids Research, 2017 https://doi.org/10.1093/nar/gkx236^42^	https://biosig.lab.uq.edu.au/mcsm_na/
DynaMut2	Rodrigues et al., Protein Sci, 2021 https://doi.org/10.1002/pro.3942^43^	https://biosig.lab.uq.edu.au/dynamut2/
SCFBio (Delhi) DNA sequence to Structure	From the Supercomputing Facility for Bioinformatics & Coputational Bioloy, IIT Delhi, Supercomputing Facility for Bioinformatics & Computational Biology, IIT Delhi (scfbio-iitd.res.in)	http://www.scfbio-iitd.res.in/software/drugdesign/bdna.jsp

### Resource availability

#### Lead contact

Further information and requests for resources and reagents should be directed to and will be fulfilled by the lead contact, Dr Daniel Vaiman (daniel.vaiman@inserm.fr)

#### Materials availability

Plasmids generated are available from the lead contact, either as DNA or to transformed bacteria, upon simple email request. Cells KO for STOX1 as well as cells overexpressing the mutant STOX1 versions are available from the lead contact upon request. In any case, a Material Transfer Agreement will be established between the provider and the recipient laboratory.

#### Data and code availability

• Data: datasets of microarray have been deposited in EBI-EMBL databases, under the numbers E-MTAB-13352 and E-MTAB-13357. They are freely available. In these databases, raw data as well as interpreted data are available. If future collaborators need specific help with statistical analyses, they may contact the lead contact that will provide help at the best of the laboratory possibilities.
• Code: There is no code generated specifically for the present study.
• Other Items: Any additional information required to reanalyze the data reported in this paper is available from the lead contact upon request. In particular the final report for the CRISPR-Cas9 Ko of STOX1 in BeWo cells is available as Data S1/GenScript Report on the production of two BeWo cell lines with STOX1 invalidated.

### Experimental model and study participant details

#### For cell lines: Growth conditions are the following

##### Cell culture

BeWo cell lines (which are from a choriocarcinoma, and thus XX, initially obtained in the laboratory from the ATCC 5 years ago, expanded and frozen.) were cultivated at 37°C in complete F-12 medium (Thermofisher Scientific, Waltham, Ma) supplemented with 10% fetal bovine serum and 1% penicillin/streptomycin. Transfected mutants that contain the Geneticin resistance plasmid were selected and maintained with geneticin 500 μg/mL (Thermofisher Scientific). Cells were treated with Forskolin (Merck, Darmstadt, Germany) at 20 μM for 3 days to induce cell fusion, according to a protocol previously described.^17^^,^^44^ Basically, a stock of Forskolin at 12mM was constituted and frozen in 100μL fractions. This was diluted 600 times when applied to the cells.

### Method details

#### Generation of *STOX1* invalidated BeWo Cell lines

Two full-allelic knockout cell lines were generated using the CRISPR-Cas9 gene editing technology (GenScript, NJ). BeWo cells were co-transfected with plasmids carrying Cas9 and a gRNA targeting STOX1 (AGCTCAGATTGTAGTAACGC). Two clones were selected and DNA sequenced to identify isogenic INDEL mutations: KO5 (+4/+16) and KO21 (+1/+1). Details on the clones are given as an Appendix 1.

#### Site-directed mutagenesis

The STOX1 mutants were successfully generated in pcDNA3.1 plasmids (Thermofisher Scientific) by site-directed mutagenesis using the QuickChange II XL kit (Agilent, Santa Clara, CA), strictly following the manufacturers’ protocol.

#### Generation of BeWo overexpressing *STOX1* variants

BeWo KO cells (passage 5–10) were co-transfected with these plasmids and with a geneticin G-418 resistance plasmid with a 1:20 ratio, according to a protocol previously described,^2^ following Invitrogen protocol. In details 60μL of Lipofectamin 2000 was diluted in 1.5 mL of OptiMem (ThermoFisher Scientific) and left 5 min at Room Temperature. In parallel, 24μg of plasmid DNA diluted in 1.5 mL OptiMem was prepared, added to the first mix, and incubated a further 20 min at room temperature. Then the mix was slowly added by drops to 1 0cm diameter dishes. Then, selection was carried out at 500μg/ml G-418 (Merck), for three weeks until resistant clones appeared, that were then collected separately, and grown systematically in F12 medium with 1% Penicillin-Streptomycin, FBS 10% and 500μg/ml G-418. Starting from the 2 independent BeWo KO lines (KO5 and KO21), we kept three lines per condition, i.e., 24 lines in total (including the three mutants and the wild-type version of STOX1).

#### Structural in silico analysis

To further understand the structural impact of the two missense variants in STOX1 and due to the lack of a crystal structure, we used the globular domain of the protein, as predicted by Alphafold^39^ (https://alphafold.ebi.ac.uk/entry/Q6ZVD7/), for in silico analysis. First, we used three docking tools to predict the protein-DNA binding interface of STOX1. For this purpose, we did three independent blind docking predictions using NPDock^38^ (https://genesilico.pl/NPDock/), HDOCK^40^ (http://hdock.phys.hust.edu.cn/) and HADDOCK 2.4 ^42^ (https://wenmr.science.uu.nl/haddock2.4/). For each tool, the input protein was the globular domain (1–191) of STOX1 and a B-DNA molecule generated using an in silico tool (http://www.scfbio-iitd.res.in/software/drugdesign/bdna.jsp) containing the DNA binding motif know for STOX1.^2^ In addition, we also used the APBS Electrostatics Plugin from PyMOL 2.5 (https://pymol.org/2/) to compute the electrostatic potential molecular surface of STOX1. After this, we used the models with the best docking scores and the highest confidence for further analysis.

As the three tools showed consistent results, we used the mCSM-NA webserver^42^ (https://biosig.lab.uq.edu.au/mcsm_na/) to predict the effects of the Y153H and T188N mutations on nucleic acid binding affinities. Finally, we used the DynaMut2 webserver^43^ (https://biosig.lab.uq.edu.au/dynamut2/) to predict the structural changes of the Y153H and T188N mutations as well as the Δ vibrational entropy energy between Wild-Type and mutant. All figures were generated in PyMOL 2.5 (https://pymol.org/2/).

#### Transcriptome analysis

100 ng of RNA were analyzed using the ClariomD microarray assay (Affymetrix, Santa Clara, CA). Library preparation, hybridization and data acquisition were performed by the GENOM‘IC platform of the Cochin Institute, according to manufacturer’s instructions. Gene and exon level expressions were processed and extracted from the ClariomD microarray using the Transcriptomic Analysis Console (TAC) 4.0 provided by Affymetrix. Gene expression levels of each cell line were normalized by the mean value of the STOX1A WT overexpressing cells (separately for KO5 and KO21) allowing us to determine the effect of the mutations on the transcriptomic profile of the mutants. The transcriptome data will be freely provided after submission to the EBI-EMBL database repository. The data on the two KO cell lines are available as E-MTAB-13352. The data on the mutant cells are available as E-MTAB-13357.

#### Quantitative RT-PCR

RNA was extracted from cells using the NucleoSpin RNA isolation kit (Macherey-Nagel, Düren, Germany). The reverse transcription was carried out using the MMLV reverse transcriptase kit of Invitrogen (Thermofisher Scientific). Real-time quantitative PCR was performed using the SensiFAST SYBR No-ROX Kit (Meridian Bioscience, Paris, France) and the rate of incorporation was monitored using the Open qPCR software v1.1.1.7 (Chai). The geometric average of the SDHA, TBP and PPIB CTs were used for normalization. The primers used to detect STOX1 are: STOX1 forward: CGGTGGGTGATGTCTTTCCA, STOX1 reverse: CACAGCAAAGAACTTCACCCA.

#### Incucyte live-cell imaging

Cells were seeded on a 96-well plate and treated with different concentrations of hydrogen peroxide (0 μM, 5 μM, 50 μM and 100 μM). The plate was placed into the IncuCyte S3 Live Cell Analysis system (Sartorius, Paris, France) and analyzed every 2 h for 5 days. CellROX Green (C10444, Thermofisher Scientific) was used to quantify reactive oxygen species and Nuclight Rapid Red Dye (Sartorius) was used to stain nuclei and assess cell proliferation. After 1 day, cells were treated with either Forskolin (20 μM) or DMSO vehicle from a stock prepared at 12 mM (600 times dilution in the medium, thus avodiing the toxic effects of DMSO that declare at more than 1%). Cells were stained with 5 μM Di-8-ANEPPS (Thermofisher Scientific) after 3.7 days.

#### Quantification of hCG-beta

Cells were plated at equal concentrations on a 6-well plate and were treated either with Forskolin 20 μM or DMSO vehicle for 4 days. The secretion of hCG was quantified analyzing the supernatants with the Human hCG-beta ELISA kit (Thermofisher Scientific), according to the manufacturer’s instructions.

#### Cell proliferation and viability assay

Cells were seeded in duplicates on two identical 96-well plates at 5000 cells per well. To test the oxidative stress management, cells were treated with either 0 or 100 μM H_2_O_2_. The first plate was then treated with 10% WST-8 with the CCK-8 kit (Merck), according to manufacturer’s instructions and incubated for 2 h at 37°C. The absorbance was then measured at 460 nm. We proceeded the same way with the second plate 4 days later. Cell viability was determined subtracting absorbance values of day one from the respective absorbance values of day four.

#### Immunofluorescence

Cells were plated on a Permanox Chamber Slide system (Merck), fixed with 4% (w/v) paraformaldehyde (PFA) and permeabilized with 0.2% Triton X-100. The cells were incubated in blocking buffer [BSA 5% (w/v) in PBS] for 30 min at room temperature, then overnight at 4°C with the primary anti-body (anti-STOX1, PA5-65193, Merck) and finally with the secondary antibody for 1 h at room temperature. Slides were mounted with Vectashield/DAPI (Vector Laboratories, Newark, CA), observed and recorded using a Nikon Eclipse E600 microscope Zeiss Axiophot epifluorescence microscope (Nikon, Tokyo, Japon). Images were digitally acquired with a camera (Coolpix 4500, Nikon) and the fluorescence measured with the ImageJ software (Java).

#### Bioinformatics

Most analyses of the transcriptome were carried out using the WebGestalt website, using the Gene Set Enrichment option rather than the Overrepresentation analysis, which depends upon the arbitrary definition of thresholds.

### Quantification and statistical analysis

In the different experiments, statistics were based on parametric tests, mostly t-test and ANOVA followed by post-hoc Student Neuman-Keuls tests using the StatistiXL add-in of Microsoft Excel, or Prism ANOVA with the Welch correction when the variances were heterogeneous; post-hoc tests, in this case, were Dunnett’s to compare the WT cells versus the mutant cells, or Tukey post-hoc test to compare all the groups by pairs. Details are presented in the legends of each figure where it is applied. In all the figures, ∗, ∗∗, ∗∗∗, correspond to p values < 0.05, 0.01 and 0.001, respectively.
