# Supplementary material for: Linking genotype to trophoblast phenotype in preeclampsia and HELLP syndrome associated with STOX1 genetic variants
Source: iScience. 2024 Feb 16;27(3):109260. doi: 10.1016/j.isci.2024.109260 (PMC10910284; doi:10.1016/j.isci.2024.109260)
Supplement: Document S1. Figures S1–S3 and Data S1 [file mmc1.pdf]

iScience, Volume *27*

Supplemental information

Linking genotype to trophoblast phenotype in preeclampsia and HELLP syndrome associated with *STOX1* genetic variants

Lorenzo Costa, Luis Bermudez-Guzman, Ikram Benouda, Paul Laissue, Adrien Morel, Karen Marcela Jiménez, Thierry Fournier, Laurence Stouvenel, Céline Méhats, Francisco Miralles, and Daniel Vaiman

Supplementary Figures

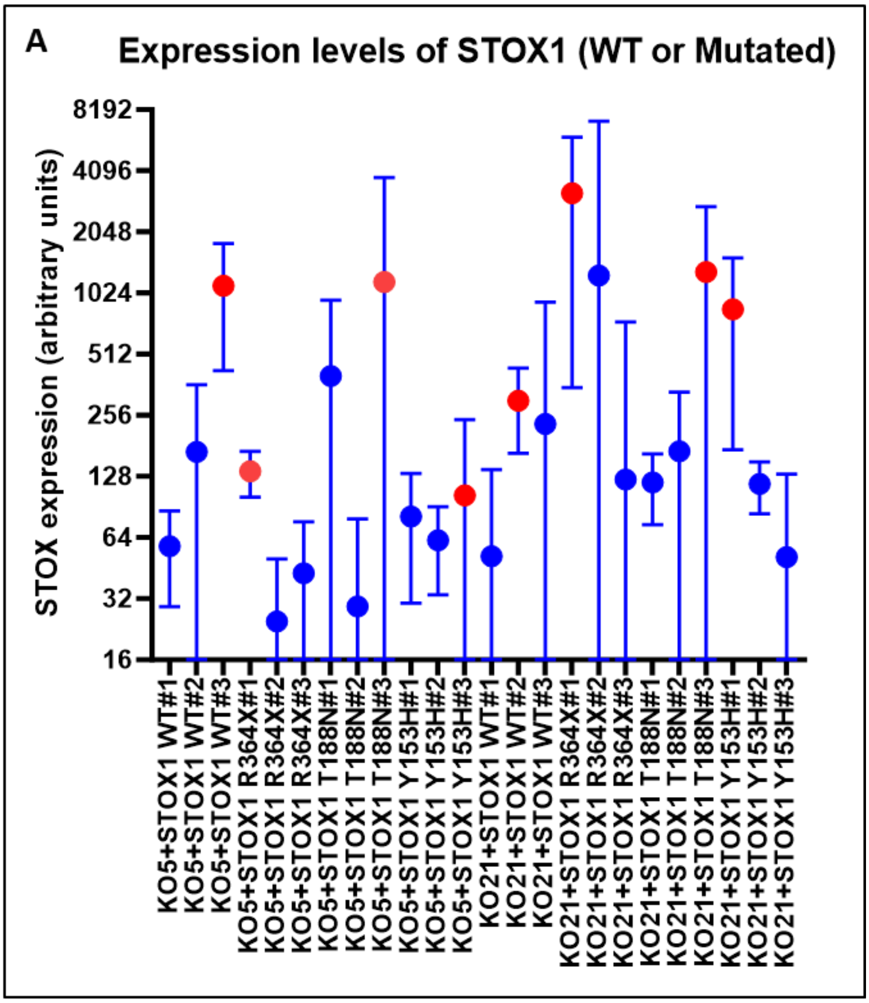

Supplementary Figure S1, related to Table 1, and STAR Methods (Generation of BeWo overexpressing *STOX1* variants) : Expression levels of STOX1 variants in the stable lines used in this study (stable transformants, maintained under geneticin G-418 welection where STOX1 (WT or mutant) was overexpressed). The expression level was estimated by RT-qPCR from two independent culture at more than 6 months interval (error bars represent Standard Deviations between the two experiments). Three clones were obtained from each KO cell line (KO5 and KO21), and for further experiments, either the 24 cells were used (transcriptome analysis), or the 8 ones having the highest STOX1 expression (red circles) were used (incucyte live cell imaging experiments).

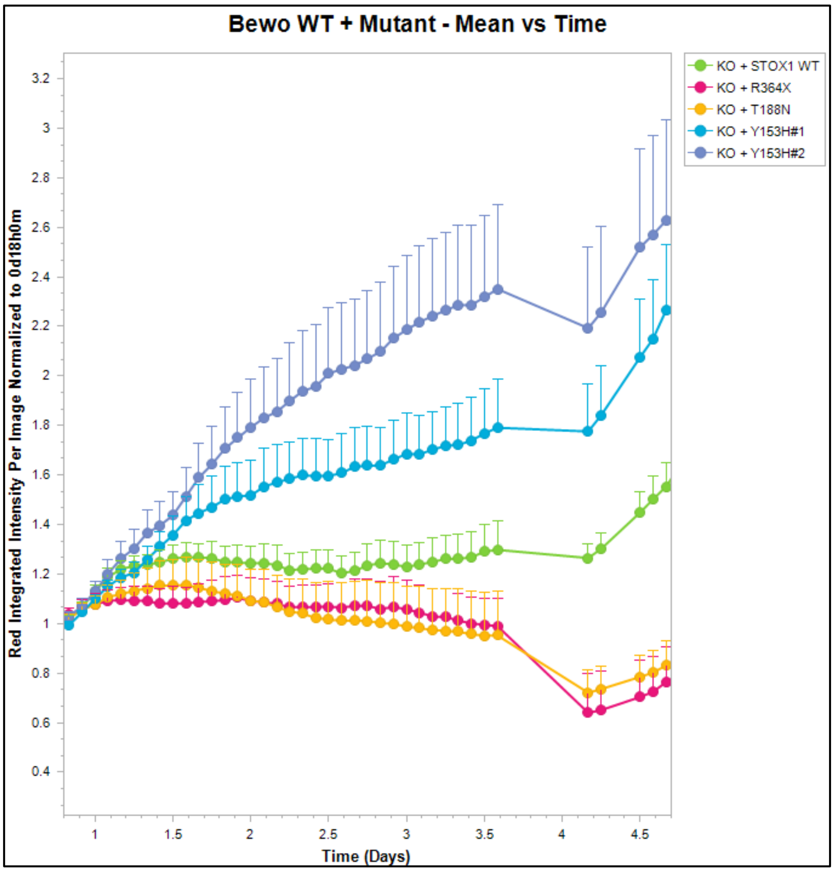

Supplementary Figure S2, related to STAR Methods (Incucyte live-cell imaging): Live cell imaging of KO cell lines were variants of the gene are overexpressed. R364X and T188N slowed down the proliferation, while Y153H (two cell lines presented) tended to increase the growth rate of the cell lines. The cells were labeled with NucLight red (Sartorius), and the surface occupied by the red fluorophore was measured as a proxy to the confluence of the cells.

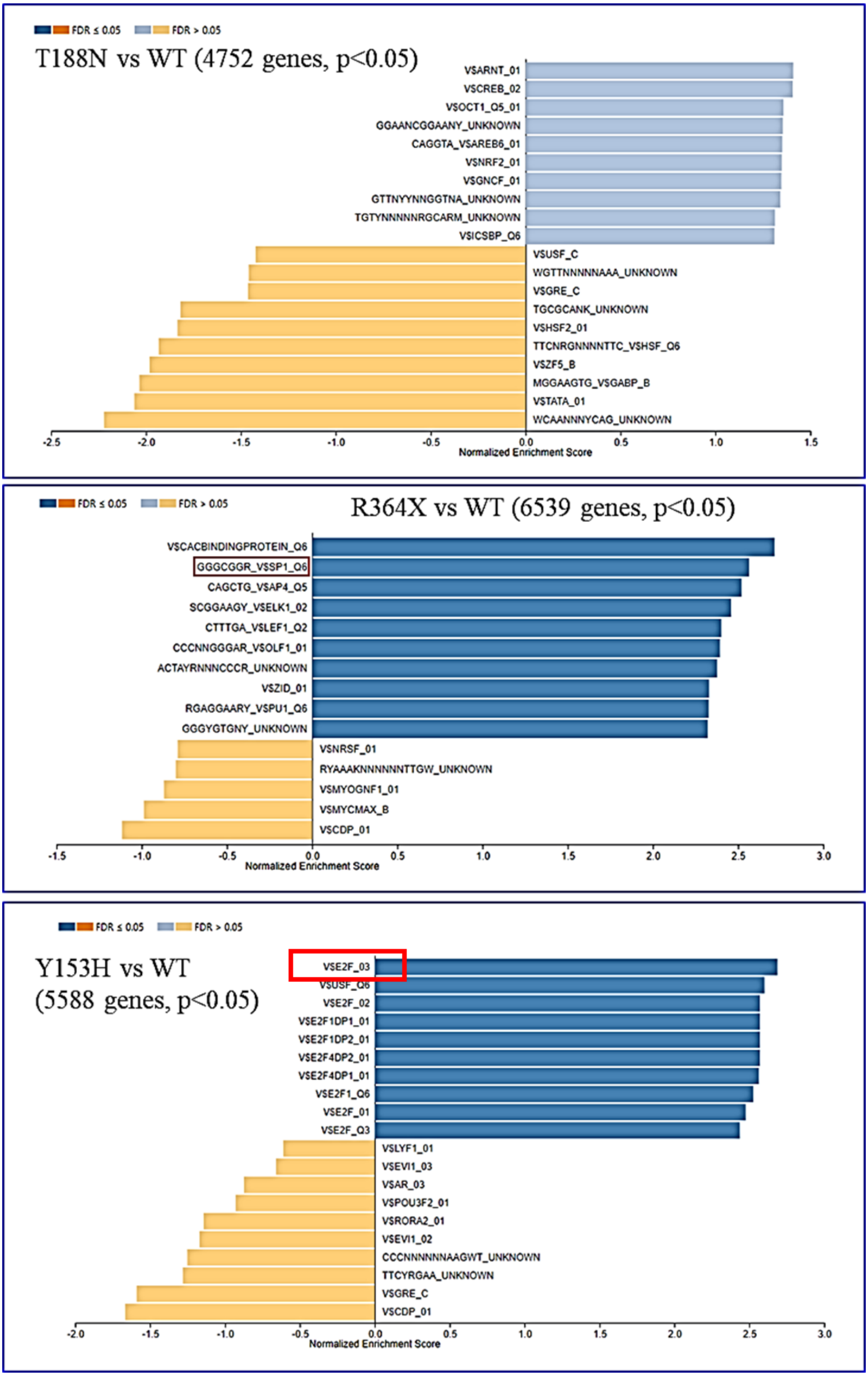

FDR ≤ 0.05
FDR > 0.05
T188N vs WT (4752 genes, p<0.05)
V$ARNT_01
V$CREB_02
V$OCT1_Q5_01
GGAANCGGAANY_UNKNOWN
CAGGTA_V$AREB6_01
V$NRF2_01
V$GNCF_01
GTTNYYNNGGTNA_UNKNOWN
TGTYNNNNNRGCARM_UNKNOWN
V$ICSBP_Q6
V$USF_C
WGTTNNNNNAAA_UNKNOWN
V$GRE_C
TGCGCANK_UNKNOWN
V$HSF2_01
TTCNRGNNNNTTC_V$HSF_Q6
V$ZF5_B
MGGAAGTG_V$GABP_B
V$TATA_01
WCAANNNYCAG_UNKNOWN
-2.5
-2.0
-1.5
-1.0
-0.5
0.0
0.5
1.0
1.5
Normalized Enrichment Score
FDR ≤ 0.05
FDR > 0.05
R364X vs WT (6539 genes, p<0.05)
V$CACBINDINGPROTEIN_Q6
GGGCGGR_V$SP1_Q6
CAGCTG_V$AP4_Q5
SCGGAAGY_V$ELK1_02
CTTTGA_V$LEF1_Q2
CCCNNGGGAR_V$OLF1_01
ACTAYRNNNCCCR_UNKNOWN
V$ZID_01
RGAGGAARY_V$PU1_Q6
GGGYGTGNY_UNKNOWN
V$NRSF_01
RYAAAKNNNNNNTTGW_UNKNOWN
V$MYOGNF1_01
V$MYCMAX_B
V$CDP_01
-1.5
-1.0
-0.5
0.0
0.5
1.0
1.5
2.0
2.5
3.0
Normalized Enrichment Score
FDR ≤ 0.05
FDR > 0.05
Y153H vs WT
(5588 genes, p<0.05)
V$E2F_03
V$USF_Q6
V$E2F_02
V$E2F1DP1_01
V$E2F1DP2_01
V$E2F4DP2_01
V$E2F4DP1_01
V$E2F1_Q6
V$E2F_01
V$E2F_Q3
V$LYF1_01
V$EVI1_03
V$AR_03
V$POU3F2_01
V$RORA2_01
V$EVI1_02
CCCNNNNNNAAGWT_UNKNOWN
TTCYRGAA_UNKNOWN
V$GRE_C
V$CDP_01
-2.0
-1.5
-1.0
-0.5
0.0
0.5
1.0
1.5
2.0
2.5
3.0
Normalized Enrichment Score

Supplementary Figure S3, related to Figure 3C: Enrichment of specific DNA binding sites detected by GSEA comparing the genes that are deregulated in the cells transfected with mutant versions of STOX1 versus WT versions. Significant enrichment (FDR<0.05) are represented by dark colors. None is detected in the T188N mutant, while the enrichment in E2F3/E2F1 binding sites was obvious following overexpression of Y153H), as represented by the red box. An enrichment of the SP1 binding site was also detected in genes modified following overexpression of the R364X mutant (box in purple).

Data S1: Report on the generation of the KO cell lines

Order U605YEF270 FINAL REPORT

Experiment Name: Development of BeWo/STOX1 Knockout Cell Line

Experiment Organization: GenScript USA Inc.

Address: 860 Centennial Ave Piscataway, NJ08854, US

Client: Universidad del Rosario

Report Date: 12/11/2019

Page Number: 14

PUBLIC

Start Date: 06/28/2019

End Date: 12/11/2019

This study was conducted according to the procedures described in this report. All data presented are authentic, accurate and correct to the best of our knowledge. Cells will be stored at GenScript only for 6 months after cells are shipped. Cell stock service can be provided upon client's request within the 6 months.

For research use only

GenScript report No. RCE20191129LR01

SIGNATURE

This report is being submitted by the following personnel.

**Post**	**Name**	**Signature**	**Date**
**Staff**	**Lynn**	Lynn	12/11/2019

**Post**	**Name**	**Signature**	**Date**
**Supervisor**	**Zhengdong Qin**	Zhengdong Qin	12/11/2019

**Post**	**Name**	**Signature**	**Date**
**Project Manager**	**Harry Wang**	Harry Wang	12/11/2019

PUBLIC

GenScript USA, Inc.
860 Centennial Ave. Piscataway, NJ 08854
Tel: 1-732-885-9188

PUBLIC

INDEX

1. Summary 5

2. Materials and Equipment 5

2.1 Materials: 5

2.2 Equipment: 5

3. Experiments and Results 5

3.1 gRNA design and validation 5

3.2 Isogenic Clone Generation and Sanger Sequencing screening 6

3.3 Characterization of knockout clones 7

3.3.1 RT-PCR 7

3.3.2 Off-target analysis 8

4. Mycoplasma test 11

5. Conclusion 11

6. Packing List 11

GenScript USA, Inc.
860 Centennial Ave. Piscataway, NJ 08854
Tel: 1-732-885-9188

7. Appendix 12

Appendix 1: Protocol for thawing and maintaining the knock-out cell lines............. 12

Appendix 2: Primers for PCR 14

Appendix 3: Map of cloning vector 15

PUBLIC

1. Summary

This report summarizes the development of a CRISPR knockout cell line by using GenCRISPR™ gene editing technology. Based on the genomic sequences on database, target gene sequence was analyzed and target sites were located according to the rules of designing a targeting guidance RNA (gRNA) for GenCRISPR™ system. By transient co-transfection of plasmids carrying the gRNA and Cas9, the endogenous target gene was targeted and mutated, resulting in consequential reduction (or removal) of the expression of the encoded protein. Isogenic knockout cell clones were generated by cultivating diluted the transfected cells in 96-well plates and were identified by Sanger sequencing screening.

2. Materials and Equipment

2.1 Materials:

Materials	Company	Cat.No.	Lot.No.
F-12K	Gibco	21127-022	2071669
FBS	Gibco	10099-141C	2109290CP
DMSO	Sigma	D2650-100ML	RNBH1690

2.2 Equipment:

Instrument	Company	Model	Fixed assets number
FACS	BD	FACSJazzTM	GR13010174
Centrifuger	Eppendorf	5804	GR17010323
Forma ClassII A2 Biological Safety Cabinet	Thermo	1285	GR17010319
Forma SeriesII Water Jacteted CO2 Incubator	Thermo	3951	GR11011128
Microscope	Shanghai optical instrument import and export co., LTD	37XC	GR16010778

3. Experiments and Results

3.1 gRNA design and validation

The gRNAs were designed to target the gene (Figure 1). The gRNA cleavage efficiency was tested in cells by transient

GenScript USA, Inc.
860 Centennial Ave. Piscataway, NJ 08854
Tel: 1-732-885-9188

PUBLIC

transfection and the gRNA with highest cleavage efficiency was selected for generation of the knockout cell line.

gRNA ID	Sequence	Cleavage efficiency*	Target gene (Genbank ID)	Cell line	Comment
T1	TCAATCTCAGTTCGTACCTT	35.9%	STOX1(ENSG00000165730)	HEK293T	Not Selected
T2	ACGAACTGAGATTGAGTTAT	25.5%	STOX1(ENSG00000165730)	HEK293T	Not Selected
T3	AGCTCAGATTGTAGTAACGC	53%	STOX1(ENSG00000165730)	HEK293T	Selected

*Cleavage efficiency is revealed by sequencing trace analysis with on-line tool TIDE (https://tide-calculator.nki.nl/)

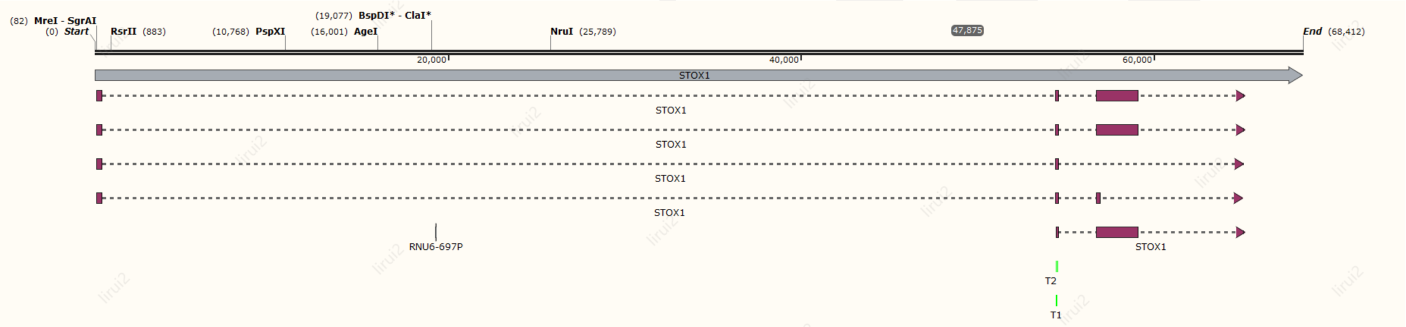

Figure 1. Map of gRNA locations
Location of gRNAs was indicated by arrows.

3.2 Isogenic Clone Generation and Sanger Sequencing screening

The gRNA was transfected in the host cells and the transfected cells were plated in 96-well plates by limit dilution to generate isogenic single clones. The clones were picked from wells and screened by DNA sequencing to identify isogenic knockout clones.

Clone ID	INDELs*	Genotype	Host Cell line	Data
Clone 5	+4/+16	Full-allelic knockout	BeWo	Figure 2
Clone 21	+1/+1	Full-allelic knockout	BeWo	Figure 2
Clone 15	0/0	Wildtype	BeWo	Figure 2

INDELs is the bps of insertion (+) or deletion (-) on alleles. “/” is used to separate INDELs among different alleles.

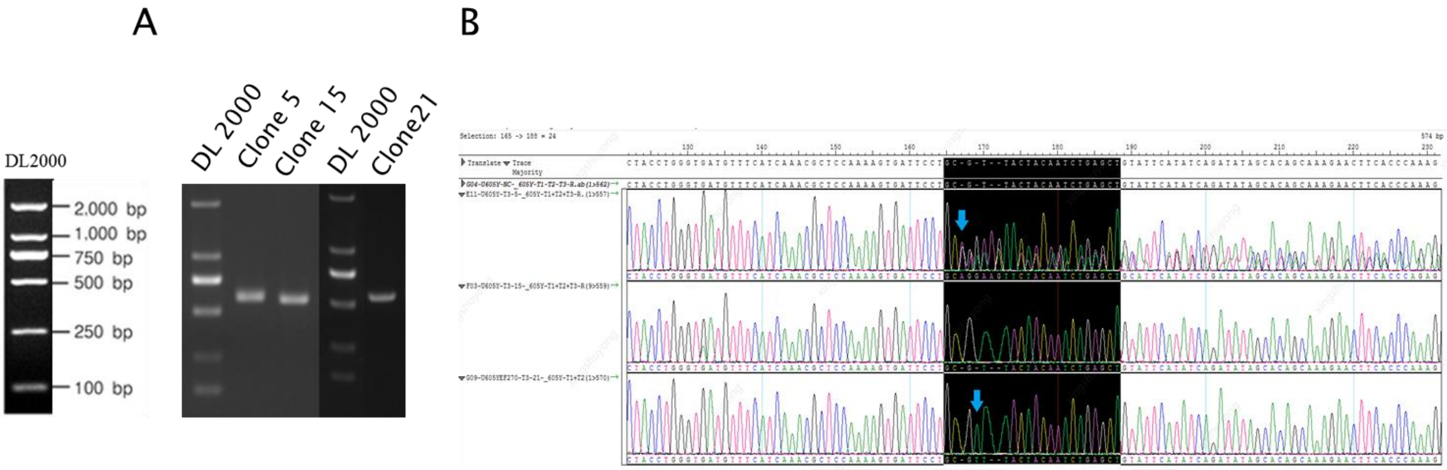

Figure 2. Genotyping of single cell clones.
A. Map of electrophoresis of PCR products. B. Alignment of sequencing diagram.
gRNA targeting site was marked in black. Arrows indicate sites of INDEL mutation.

3.3 Characterization of knockout clones

3.3.1 RT-PCR

The total RNA was extracted from knockout clones and reverse-transcribed to cDNA. Specific primers flanking the gRNA targeting site on cDNA were used to amplify the DNA and followed by Sanger sequencing.

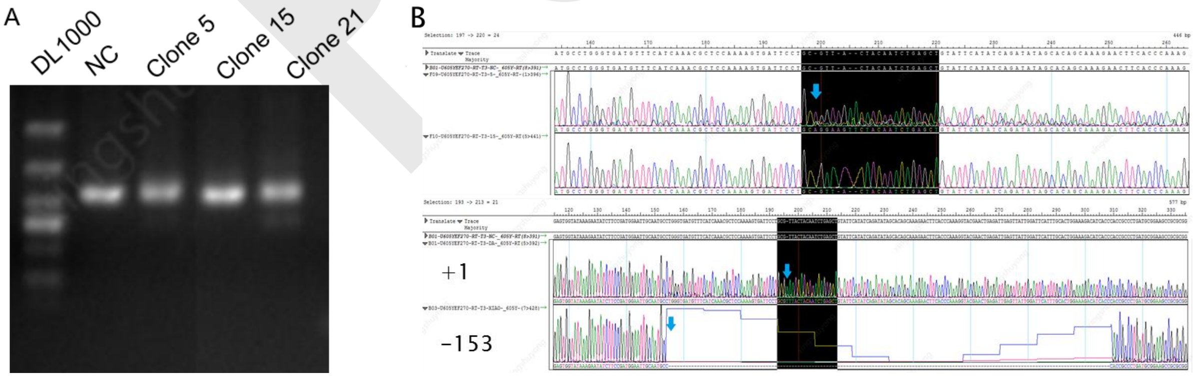

Figure 3. Genotyping of single cell clones.

A. Map of electrophoresis of RT-PCR products. B. Alignment of sequencing diagram.

gRNA targeting site was marked in black. Arrows indicate sites of INDEL mutation.

3.3.2 RT-qPCR

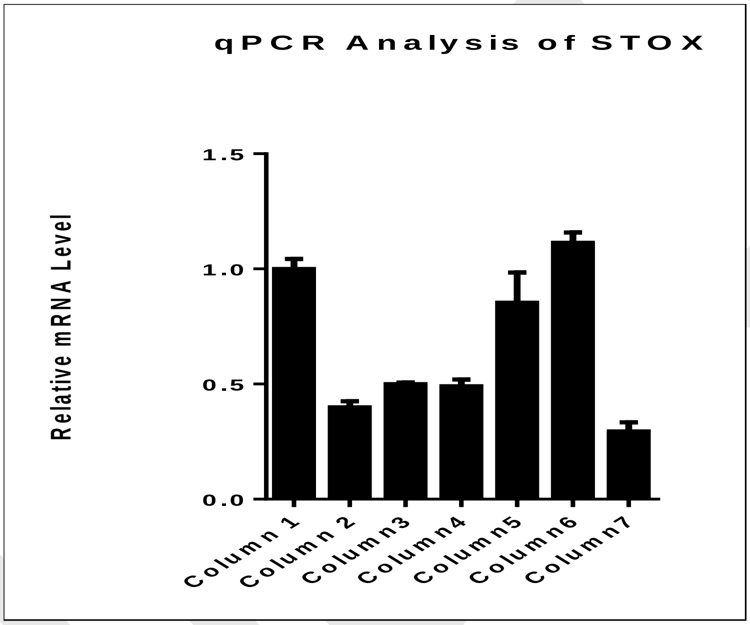

Figure 4. qPCR analysis of STOX1 in BeWo/STOX1 host cell and clone 5, 6, 7, 14, 15 and 21

Column 1: BeWo host cell;

Column 2: BeWo-STOX1-clone5;

Column 3: BeWo-STOX1-clone6;

Column 4: BeWo-STOX1-clone7;

Column 5: BeWo-STOX1-clone14;

Column6 : BeWo-STOX1-clone15;

Column 7: BeWo-STOX1-clone21

3.3.3 Off-target analysis

GenScript report No. RCE20191129LR01

The following potential off-target sites given by gRNA design tool (CCTOP) were listed in the below table and sequenced by Sanger sequencing. It was revealed none of off-target sites was edited by the gRNA.

Coordinates	strand	MM	target_seq	PAM	distance		gene name	gene id
chr10:68882052-68882074	+	0	AGCTCAGA[TTGTAGTAACGC]	AGG	0	E	STOX1	ENSG00000165730
chr5:164601724-164601746	+	4	A**C**CTCA**AC**[TTG**G**AGTAACGC]	TGG	0	E	CTC-340A15.2	ENSG00000241956
chr4:1678209-1678231	-	3	AGCT**AG**GA[TTGTAGT**C**ACGC]	AGG	5189	I	Y_RNA	ENSG00000207009
chr11:96262841-96262863	-	3	AGCT**G**AGA[TT**A**TAGTAA**A**GC]	AGG	78520	I	MAML2	ENSG00000184384
chr7:93562737-93562759	-	4	AGC**CA**AGA[TT**A**TAGTAA**T**GC]	AGG	2927	I	CALCR	ENSG00000004948
chr3:51952385-51952407	+	4	AGCT**T**A**C**A[TT**C**TAGTAA**A**GC]	AGG	0	E	RP11-155D18.13	ENSG00000280422

Figure 5. Top 5 potential off-target sites of gRNA T3

PUBLIC

GenScript USA, Inc.
860 Centennial Ave. Piscataway, NJ 08854
Tel: 1-732-885-9188

GenScript report No. RCE20191129LR01

CTC-340A15.2	1 -><br>2 -><br>3 -><br>4 ->	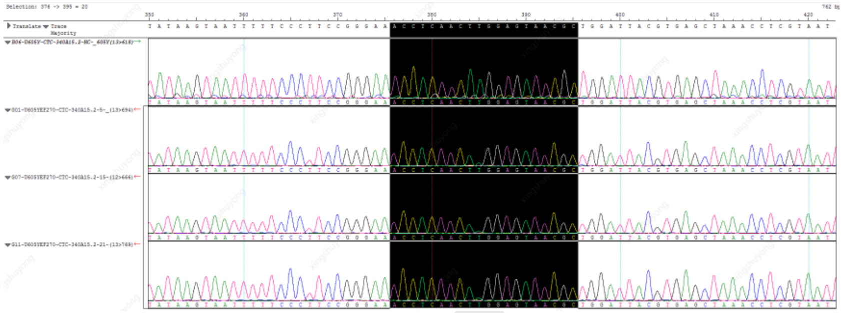
Y_RNA	1 -><br>2 -><br>3 -><br>4 ->	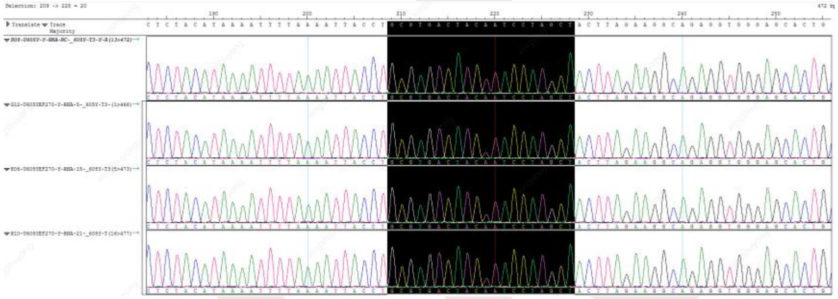
MAML2	1 -><br>2 -><br>3 -><br>4 ->	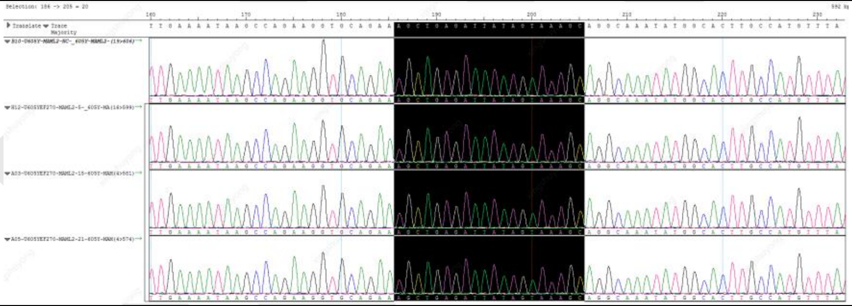
CALCR	1 -><br>2 -><br>3 -><br>4 ->	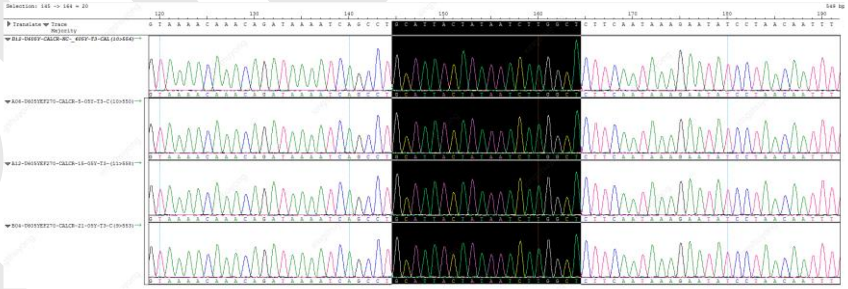
RP11-155D18.13	1 -><br>2 -><br>3 -><br>4 ->	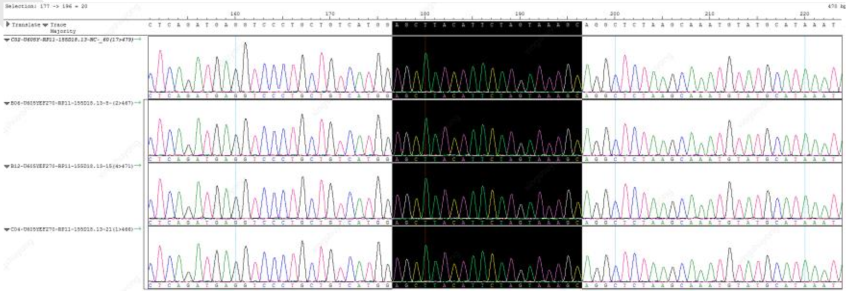

Figure 6. Off-target analysis by Sanger sequencing

GenScript USA, Inc.
860 Centennial Ave. Piscataway, NJ 08854
Tel: 1-732-885-9188

1：NC; 2: Clone5; 3: Clone15; 4: Clone 21

4. Mycoplasma test

The mycoplasma test was performed with MycoAlert™ PLUS Mycoplasma Detection Kit of Lonza.

Cell	Value	Result*
Clone 5	0.71	Passed
Clone 21	0.86	Passed
Clone 15	0.63	Passed

*The test has been designed to give ratios of less than 1 with uninfected samples and routinely produce ratios greater than 1 for samples infected with mycoplasma.

5. Conclusion

BeWo/STOX1 knockout Clone 5 and 21 and wild type Clone 15 were successfully generated and tested mycoplasma-free for delivery.

6. Packing List

Order U605YEF270

Cell lines (Shipping Condition: -80°C Dry Ice, Store at -196°C)

Note: please thaw and culture the cells following the protocol（Appendix 1） in the report.

Name: BeWo/STOX1 INDEL:+1/+1 Clone 21
Quantity: 1x10^6cells/vial
Lot No.: U605YEF270-12/P2EK003
Number of vial: 2 vials
Store at：-196°C

Name: BeWo/STOX1 INDEL:+4/+16 Clone 5
Quantity: 1x10^6cells/vial

PUBLIC

GenScript report No. RCE20191129LR01
Lot No.: U605YEF270-12/P2EJ005
Number of vial: 2 vials
Store at: -196°C

Name: BeWo/STOX1 INDEL:0/0 Clone 15
Quantity: 1x10^6cells/vial
Lot No.: U605YEF270-12/P2EJ002
Number of vial: 2 vials
Store at: -196°C

Plasmid（Shipping Condition: Room temperature, Store at -20°C）

Name: U605YEF270_STOX1_T1 in pSpCas9(BB)-2A-GFP(PX458)
Quantity: 4µg
Lot No.: C869JEG030-1 / G67444
Number of vial: 1 tube
Store at -20 °C

Name: U605YEF270_STOX1_T2 in pSpCas9(BB)-2A-GFP(PX458)
Quantity: 4µg
Lot No.: C869JEG030-3 / G67448
Number of vial: 1 tube
Store at -20 °C

Name: U605YEF270_STOX1_T3 in pSpCas9(BB)-2A-GFP(PX458)
Quantity: 4µg
Lot No.: C869JEG030-5 / G67456
Number of vial: 1 tube
Store at -20 °C

7. Appendix

Appendix 1: Protocol for thawing and maintaining the knock-out cell lines

To insure the highest level of viability, thaw the vial and initiate the culture as soon as possible upon receipt. If upon arrival, continued storage of the frozen culture is necessary, it should be stored in liquid nitrogen vapor

PUBLIC

phase and not at —80°C. Storage at —80°C will result in loss of viability.

Recommended Cell Culture Medium: F-12K, 10% FBS

Freeze Medium: 95% complete growth medium, 5% (V/V) DMSO

Thawing cells:

- Transfer 5ml of complete growth medium to a 15ml Centrifuge Tube.
- Remove the cryovial containing the frozen cells from liquid nitrogen storage and immediately place it into a 37°C water bath.
- Quickly thaw the cells (< 1 minute) by gently swirling the vial in the 37°C water bath until the cryovial completely thawed.
- Transfer the vial into a laminar flow hood. Before opening, wipe the outside of the vial with 75% ethanol.
- Transfer the thawed cells to the 15ml Centrifuge Tube that containing 5ml of complete growth medium.
- Centrifuge the cell suspension at 800rpm for 5 minutes.
- After the centrifugation, decant medium, and gently re-suspend the cells in 10ml complete growth medium, and transfer them into 6cm dish and incubate at 37°C, 5% CO_2.

Maintaining cells:

- For adherent cells:
  1. Volumes are given for 10 cm petri dish
  2. Remove and discard culture medium.
  3. Briefly rinse the cell layer with 0.25% (w/v) Trypsin- 0.53 mM EDTA solution to remove all traces of serum which contains trypsin inhibitor.
  4. Add 1.5 mL of Trypsin-EDTA solution to 10 cm petri dish and observe cells under an inverted microscope until cell layer is dispersed (usually within 5 to 15 minutes).
  5. Add 3.5 mL of complete growth medium and aspirate cells by gently pipetting.

GenScript USA, Inc.
860 Centennial Ave. Piscataway, NJ 08854
Tel: 1-732-885-9188

PUBLIC

6. Add appropriate aliquots of the cell suspension to new culture vessels.
7. Incubate cultures at 37°C.

Appendix 2: Primers for PCR

Appendix 1: Primers for genotyping

Primers for *STOX1* gRNA T3 site (5'>3'):

Forward: ATTGCATGACCTCCTGAACTCT

Reverse: TGATGCAGGACTTCTTCTGGC

Primers for RT-PCR (5'>3'):

Forward: GCTGGCGCTAGTGCTGTG

Reverse: CTGGCATCAGGCGACTTTCA

Primers for RT-qPCR (5'>3'):

Forward: CCACTCTGGGAACGCTGATTA

Reverse: CTGGCATCAGGCGACTTTCA

PUBLIC

GenScript report No. RCE20191129LR01

Appendix 3: Map of cloning vector

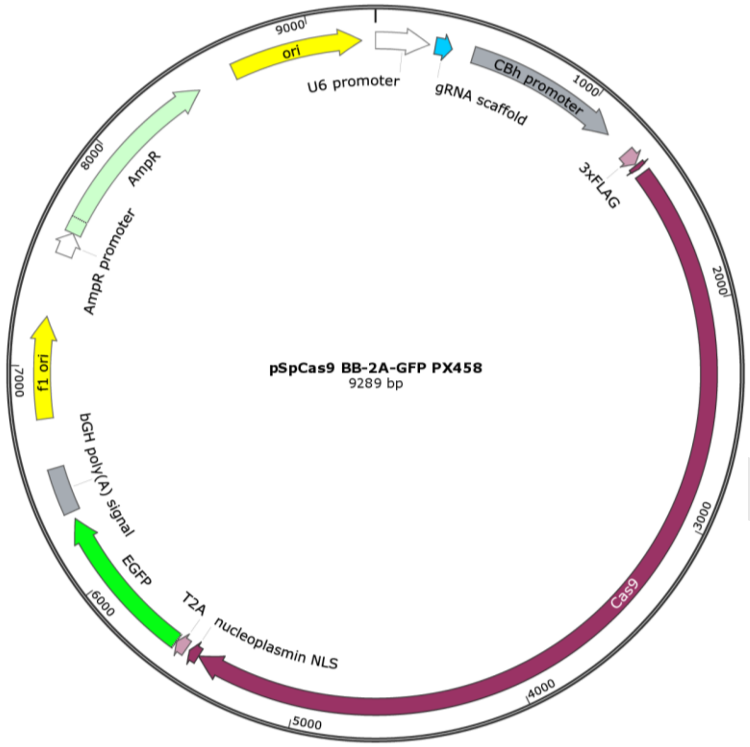

GenScript USA, Inc.
860 Centennial Ave. Piscataway, NJ 08854
Tel: 1-732-885-9188
